# Synthesis of Perovskite Nanowires and Their Application for Photodetectors

**DOI:** 10.1002/advs.202510428

**Published:** 2025-08-23

**Authors:** Jiajun Guo, Chenyuan Wang, Xin Zhao, Gangjian Hu, Xinren Zhang, Jizhong Jiang, Wei Wei, Liang Shen

**Affiliations:** ^1^ State Key laboratory of integrated Optoelectronics College of Electronic Science and Engineering International Center of Future Science Jilin University Changchun 130012 P. R. China; ^2^ Westlake Institute for Optoelectronics Fuyang Hangzhou 311421 P. R. China

**Keywords:** application, nanowires, perovskite, photodetectors, synthesis

## Abstract

1D nanowires (broadly including microwires and quantum wires) of metal halide perovskites exhibit several unique properties due to their distinctive morphology, such as enhanced responsivity under weak light, mechanical flexibility, and optical anisotropy. These perovskite nanowires can be readily fabricated into lateral devices as photoconductive photodetectors. Herein, the synthesis of perovskite nanowires and their applications for photodetectors are primarily focused on, providing an overview of the major synthesis strategies for perovskite nanowires (and arrays) since their initial reports, and analyzing how structural design and modification strategies can improve the performance of nanowire‐based devices. The performance of some reported nanowire photodetectors is summarized and analyzed. In addition, the applications for nanowire photodetectors in polarization detection, flexible sensing, and image sensing, followed by a conclusion and outlook are discussed in detail.

## Graphical abstract

1D metal halide perovskite nanowires possess unique properties such as high responsivity under weak light, flexibility, and optical anisotropy. This work reviews key synthesis strategies for perovskite nanowires and arrays, explores structural design and modification approaches to enhance device performance, summarizes representative device metrics, and discusses their applications in polarization detection, flexible sensing, and imaging.

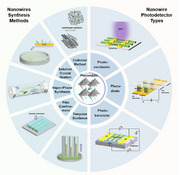

## 1 Introduction

**Figure 1:**
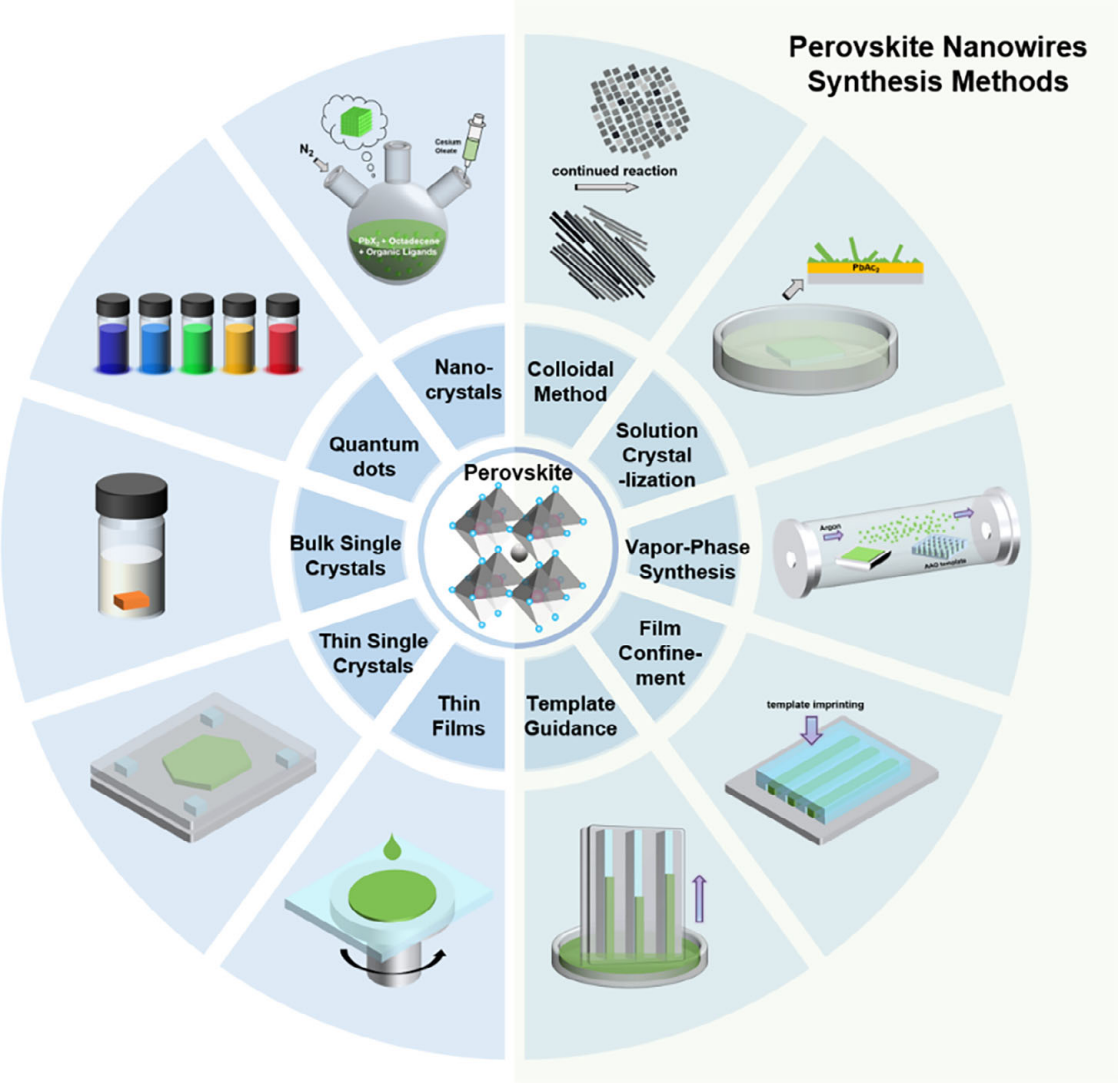
Perovskites with different morphologies and corresponding schematic diagrams of their fabrication.

The chemical formula of metal halide perovskites is ABX_3_, where the A‐site cation can be Cs^+^, methylammonium (CH_3_NH_3_^+^, MA^+^), or formamidinium (CH(NH_2_)_2_^+^, FA^+^), the B‐site is occupied by metal cations such as Pb^2+^ or Sn^2+^, and X represents halide anions such as I^−^, Br^−^, and Cl^−^. In an ideal perovskite crystal structure, the B cations and X anions are coordinated to form a halide octahedral structure, denoted as [BX_6_]^4−^, with X at the vertices and the B cations positioned at the center of the octahedron. Adjacent octahedra are connected through shared corner vertices, forming a 3D network framework with the A cations occupying the gaps between the octahedra. Perovskites exhibit a range of excellent properties, such as high light absorption coefficient,^[^ ^1^, ^2^ ^]^ long carrier diffusion length,^[^ ^3^, ^4^ ^]^ high carrier mobility, extended carrier lifetime,^[^ ^5^ ^]^ low defect density,^[^ ^6^ ^]^ and a tunable bandgap that spans the entire visible light spectrum.^[^ ^7^ ^]^ Additionally, they can be synthesized through simple and diverse fabrication methods. Due to these advantages, perovskite materials have emerged as outstanding candidates for next‐generation high‐performance, low‐cost optoelectronic devices, with widespread applications in solar cells,^[^ ^8^, ^9^ ^]^ photodetectors (PDs),^[^ ^10^, ^11^, ^12^ ^]^ radiation detectors,^[^ ^13^, ^14^ ^]^ image sensors,^[^ ^15^ ^]^ light‐emitting diodes (LEDs),^[^ ^16^, ^17^ ^]^ lasers,^[^ ^18^ ^]^ and other optoelectronic devices. Various morphologies of perovskites have been synthesized, including bulk single crystals,^[^ ^19^ ^]^ thin single crystals,^[^ ^20^ ^]^ thin films,^[^ ^21^ ^]^ nanowires (NWs),^[^ ^22^ ^]^ nanocrystals (NCs),^[^ ^23^ ^]^ and quantum dots (QDs),^[^ ^24^ ^]^ each possessing unique characteristics. (**Figure** **1**)

NWs possess many unique and superior properties compared to other perovskite morphologies and have already found widespread applications.^[^ ^25^, ^26^, ^27^, ^28^, ^29^, ^30^, ^31^ ^]^ Specifically, NWs feature a low number of grain boundaries and a reduced dimensionality of the active region, which limit the carrier transport path and shorten the carrier transit time^[^ ^32^ ^]^ Additionally, due to their large specific surface area, the presence of surface states forms energy band structures favorable for exciton dissociation while trapping photogenerated carriers, thereby reducing their recombination probability, especially under low incident power.^[^ ^33^, ^34^ ^]^ This effect significantly extends the photogenerated carrier lifetime and enhances the ability to detect weak light. NWs possess a high aspect ratio, contributing to their excellent bending resistance, making them ideal for integration into flexible devices.^[^ ^35^ ^]^ They also exhibit axial and radial optical anisotropies, making them applicable for polarization emission and detection.^[^ ^36^ ^]^ Compared to randomly distributed NWs, nanowire arrays (NWA) show a higher optical response due to the reduced number of cross junctions and shortened carrier transport paths. When NWs are arranged in aligned arrays at well‐defined positions, current output and active area can be enhanced, while better uniformity and repeatability are achieved compared to randomly distributed NWs. The responsivity of perovskite NW‐based PDs has been shown to exceed 10^6^ A W^−1^,^[^ ^37^ ^]^ while the specific detectivity has been reported to surpass 10^15^ Jones.^[^ ^32^, ^38^, ^39^ ^]^ demonstrating their immense potential in the field of photodetection.

In this review, we summarize the primary synthesis methods and performance enhancement strategies for metal halide perovskite NWs and arrays since they were reported, as well as their performance parameters and applications for PDs. In Section 2, we introduce the synthesis of perovskite NWs and arrays, categorizing them based on synthesis approaches into template‐free and template‐assisted methods. Section 3 outlines modification strategies employed to enhance NW performance, including the construction of heterojunction (HJT), passivation or encapsulation using additives, and optimization through material selection. Section 4 provides a detailed overview of the performance testing of NW PDs, summarizing and comparing the performance of several representative devices. In Section 5, we discuss the applications of NW detectors for polarization detectors, flexible devices, and image sensors, while Section 6 offers a conclusion and outlook.

## 2 Methods for the Synthesis of Nanowires (Arrays)

Since the use of templates often plays a critical role in determining whether NWs form an ordered array, the fabrication methods are first categorized into template‐free and template‐assisted methods based on the objective of synthesis, and are further subdivided based on specific synthetic strategies. **Table** **1** provides a summary of common NW synthesis methods and lists several studies based on these approaches. Some representative examples will be introduced in the following sections.

**Table 1:** NW synthesis methods.

		Typical process	Refs.
Solution crystallization	Slip‐coating	Relative sliding of two precursor‐clamped glass slides	[37, 40]
	Drop‐coating	Drop precursor droplets on a wettability‐treated substrate, followed by evaporation	[41, 42, 43, 44, 45, 46, 47]
	Dip‐coating	Immersion of PbAc_2_ thin film in MAI solution for surface‐initiated solution growth	[34, 48, 49, 50, 51, 52, 53, 54, 55, 56, 57, 58, 59, 60, 61, 62, 63, 64]
	Spin‐coating	Two‐step sequential spin‐coating of precursors or one‐step spin‐coating	[65, 66, 67, 68, 69, 70, 71, 72, 73, 74]
	Blade‐coating	Blade‐dragging of precursor across heated plate	[35, 75, 76, 77, 78, 79]
	Antisolvent‐assisted	Precursor solution precipitates under the antisolvent vapor atmosphere	[80, 81, 82, 83, 84, 85, 86, 87]
Colloidal method	Hot‐injection method mainly	Hot‐injection of cesium oleate into lead halide in the presence of organic ligands to form NCs, followed by transformation into NWs	[88, 89, 90, 91, 92, 93, 94, 95, 96, 97, 98, 99, 100, 101, 102, 103, 104, 105]
Vapor‐phase synthesis	Epitaxial growth	Epitaxial growth on substrates such as Si, mica, and sapphire via chemical vapor deposition	[106, 107, 108, 109, 110, 111, 112, 113, 114, 115, 116, 117, 118, 119, 120, 121, 122, 123, 124, 125, 126, 127, 128, 129, 130]
	Gas–liquid–solid methods	Supersaturation of precursor vapor within nanoscale Pb droplets triggers NW growth	[131, 132, 133, 134]
Patterning method	Patterned thin film mainly	Direct patterning of perovskite thin film through photolithography and etching	[135, 136, 137, 138, 139, 140, 141, 142, 143]
Film Confinement	Imprinted thin film	Confined growth of thin films via template imprinting	[144, 145, 146, 147, 148, 149, 150, 151, 152, 153, 154, 155, 156, 157]
Template Guidance	AAO‐guided	Directing the precursor into the pores of porous anodic aluminum oxide (AAO) to form vertically aligned NWs.	[158, 159, 160, 161, 162, 163, 164, 165]
	Microfluidic	Injecting the precursor into the nanochannels of the microfluidic device	[166, 167]
	Capillary‐force‐guided	Driving the precursor into the microchannel template via capillary force	[168, 169, 170, 171, 172, 173, 174, 175]
	Wettability‐guided	Asymmetric‐wettability topographical templates enabling selective solution distribution on hydrophilic regions	[32, 36, 38, 39, 176, 177, 178, 179, 180, 181, 182, 183]
	Antisolvent‐assisted	Template‐guided antisolvent‐assisted crystallization	[184]
Template‐assisted vapor‐phase synthesis	AAO‐guided mainly	Porous AAO membrane‐templated vapor–solid–solid reaction between MAI vapor and Pb nanoclusters	[22, 185, 186, 187, 188, 189, 190, 191, 192, 193, 194, 195, 196, 197, 198, 199]

Notably, 1D perovskite structures are categorized based on their minimum linewidth: structures with a linewidth above 1 µm are referred to as microwires (MWs), those in the 100 nm to 1 µm range fall into the submicron scale, structures with tens of nanometers in linewidth are considered nanowires, and those with linewidths of only a few nanometers are termed quantum wires (QWs). However, in accordance with prevailing conventions in the literature, structures with a linewidth as small as the submicron scale can also be called NWs.

### 2.1 Template‐Free Methods

The template‐free method refers to approaches that do not rely on artificially fabricated templates, in contrast to template‐assisted strategies. Due to the absence of ordered guiding structures, the resulting perovskite NWs are often randomly interwoven and overlapped, or are synthesized as individual NWs. These methods are primarily categorized into solution‐based and vapor‐phase methods, with solution‐based methods roughly divided into solution crystallization and colloidal methods. Their main advantage is simplicity, as they typically require neither expensive equipment nor high temperatures. These methods also offer high yields and greater flexibility in substrate selection. However, NWs synthesized via colloidal methods are rarely applied in PDs, while most solution crystallization approaches are preferred. Methods such as drop‐coating and dip‐coating offer low‐cost processing and are highly convenient for investigating material properties. In addition, large‐area coating techniques like blade‐coating and spin‐coating hold great potential for commercial applications. Vapor‐phase methods typically produce NWs with higher crystal quality and lower defect density. However, it often requires high‐temperature deposition on specific substrates, resulting in relatively higher costs. Another template‐free approach involves the direct patterning of perovskites to produce precisely controlled, custom‐designed NW grating structures or individual NWs. This method is expected to play a crucial role in many specialized applications, though it requires the support of advanced micro/nanofabrication equipment.

#### 2.1.1 Solution Crystallization

**Figure 2:**
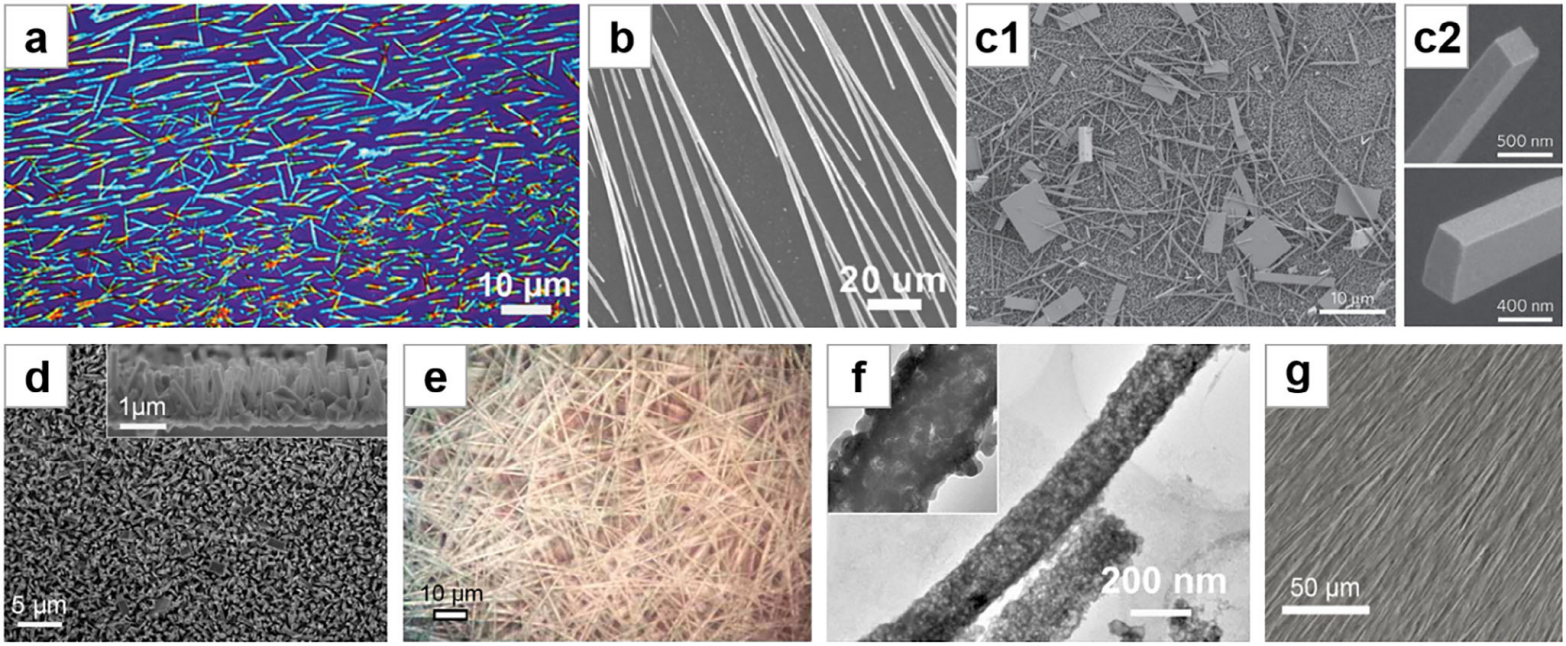
Solution crystallization method for NWs growth (I). a) Optical microscopy image of filiform crystallites grown by slip‐coating. Reproduced with permission.^[^ ^40^ ^]^ Copyright 2014, American Chemical Society. b) SEM image of NWs grown by drop‐coating. Reproduced with permission.^[^ ^42^ ^]^ Copyright 2015, American Chemical Society. c) c1: SEM image of MAPbI_3_ nanostructures grown from PbAc_2_ thin film by dip‐coating. c2: Magnified SEM images of NWs. Reproduced with permission.^[^ ^48^ ^]^ Copyright 2015, Springer Nature Limited. d) Top‐view SEM image of MAPbBr_3_ vertically aligned nanorod array. Reproduced with permission.^[^ ^60^ ^]^ Copyright 2015, American Chemical Society. e) Optical microscope image of the MAI‐PbI_2_‐DMF adduct intermediate “white” phase. Reproduced with permission.^[^ ^61^ ^]^ Copyright 2017, American Chemical Society. f) TEM image of MAPbBr_3_ NWs with porous structure. Reproduced with permission.^[^ ^62^ ^]^ Copyright 2015, John Wiley and Sons. g) SEM image of MAPbI_3_ MWs formed by dip‐coating. Reproduced with permission.^[^ ^64^ ^]^ Copyright 2018, John Wiley and Sons.

Dendrite perovskite crystals are easily formed in perovskite thin films, indicating that perovskite crystallites tend to grow uniformly along one of the crystallographic directions. As a result, using solution crystallization to precipitate perovskite NWs is a common preparation method. According to the operation, this method can be divided into techniques such as slip‐coating, drop‐coating, dip‐coating, spin‐coating, blade‐coating, and antisolvent‐assisted crystallization. Horváth et al.^[^ ^40^ ^]^ used the slip‐coating method to observe, for the first time, 1D organic lead halide perovskite in 2014. N,N‐Dimethylformamide (DMF) solution of MAPbI_3_ was placed between two glass slides and subjected to relative sliding. Upon exposure to air, the solvent evaporated and crystallized to form NW networks (**Figure** **2**a). The authors fabricated the first perovskite NW‐based PD. Although the responsivity was only 5 mA W^−1^, they explored the feasibility of anisotropic growth. Building on this, Spina et al.^[^ ^37^ ^]^ combined the slip‐coated NWs with a single‐layer graphene field‐effect transistor (FET) fabricated by chemical vapor deposition (CVD). The constructed PD achieved a responsivity of 2.6 × 10^6^ A W^−1^ at 0.65 nW mm^−2^ incident optical power and could even be used for single‐photon detection.

Deng et al.^[^ ^41^ ^]^ dropped the perovskite precursor solution onto UV–ozone‐treated substrate, where the solution was evenly distributed and naturally evaporated at room temperature to form NWs. The controllable distribution of NWs was achieved by restricting the wettability treatment to specific areas using a mask, and the growth direction of the NWs was partially guided by the inclination of the substrate. A comparison between NWs grown by drop‐coating and those formed into NW networks through an additional spin‐coating step reveals that the NW networks exhibit superior uniformity, transparency, and flexibility.^[^ ^42^ ^]^ (Figure 2b). Many additional studies have been conducted based on the simple drop‐coating method.^[^ ^43^, ^44^, ^45^, ^46^, ^47^ ^]^

In the landmark work on surface‐initiated solution growth reported by Zhu et al.,^[^ ^48^ ^]^ PbAc_2_⋅3H_2_O was drop‐coated onto the substrate and dried to form a PbAc_2_ thin film, which was then immersed face‐up in isopropanol (IPA) solution of MAI. As the low‐concentration Pb precursor slowly released from the solid PbAc_2_ film on the substrate, single‐crystal MAPbI_3_ NWs were formed after 20 h of reaction (Figure 2c). The authors analyzed that the catalyst‐free anisotropic growth of these lead halide perovskite NWs is probably driven by screw dislocations. The NWs were transferred by a simple dry contact process, demonstrating low laser threshold and high‐quality factor, with photoluminescence quantum yield (PLQY) approaching 100%. Further research based on this immersion synthesis method has been conducted.^[^ ^34^, ^49^, ^50^, ^51^, ^52^, ^53^ ^]^ Fu et al.^[^ ^54^ ^]^ from the same group first reported the synthesis of FA‐based perovskite NWs, which exhibited better optical stability and a broader tunable wavelength range compared to MA‐based perovskite NWs. Eaton et al.^[^ ^55^ ^]^ immersed PbI_2_ film into CsBr–methanol solution with mild heating to synthesize CsPbBr_3_ NWs. They demonstrated the laser emission from all‐inorganic perovskite NWs for the first time. This method has been widely extended.^[^ ^56^, ^57^, ^58^ ^]^ This group also described the synthesis of CsSnI_3_ NWs by immersing a substrate with SnI_2_ particles in CsI/2‐propanol solution.^[^ ^59^ ^]^ Wong et al.^[^ ^60^ ^]^ suspended PbAc_2_ film face down in a centrifuge tube, added MABr/IPA solution, and formed vertically aligned nanorod arrays after a long reaction. (Figure 2d). Petrov et al.^[^ ^61^ ^]^ immersed PbI_2_ film in a mixed solution of MAI in IPA and DMF, and obtained MAPbI_3_ NWs after annealing. During the synthesis, a special intermediate “white” phase crystallized in the form of 1D crystals, which were determined to be MAI‐PbI_2_‐DMF adducts (Figure 2e). The authors suggested that the NW morphology was that of the adducts, which retained their original crystal form when converted to perovskite. Therefore, the perovskite NWs appeared to be a pseudomorph. Tong et al.^[^ ^200^ ^]^ reported a method to synthesize lead‐containing NWs using cysteine, lead nitrate, and ethanolamine. Expanding on this, Zhuo et al.^[^ ^62^ ^]^ added the lead‐containing precursor NWs to the solution containing excess HBr and MABr, where they underwent an in situ conversion to MAPbBr_3_ from the outer to the inner layers, forming unique porous structures due to the dissolution and release of the organic components in the precursor (Figure 2f). Yang et al.^[^ ^63^ ^]^ used a similar method, where Pb(OAc)_2_ was dropped into an aqueous solution containing polyvinylpyrrolidone (PVP) ‐modified upconversion nanoparticles (UCNPs) and _L_‐cysteine. The formed lead‐containing NWs encapsulated the UCNPs and were further converted into perovskite NWs by being treated with a mixture of MABr and HBr, synthesizing a composite material of UCNPs and perovskite NWs. Chen et al.^[^ ^64^ ^]^ directly immersed a lyophilic‐treated substrate into perovskite precursor solution, and by pulling it out at a controlled rate, they synthesized MWs with better orientation (Figure 2g).

**Figure 3:**
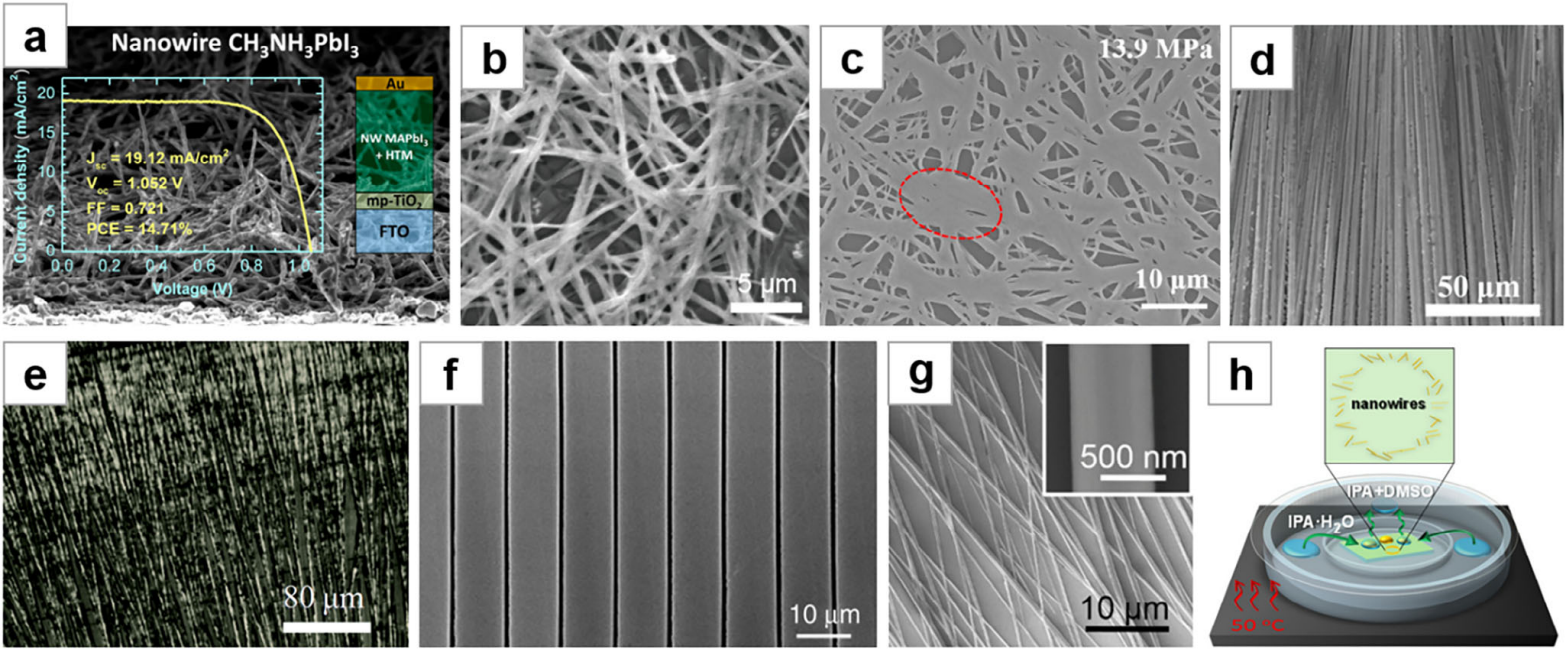
Solution crystallization method for NWs growth (II). a) MAPbI_3_ NWs grown by spin‐coating to fabricate solar cells. Reproduced with permission.^[^ ^65^ ^]^ Copyright 2015, American Chemical Society. b) MAPbI_3_ thin films form NWs through dissolution–recrystallization process. Reproduced with permission.^[^ ^66^ ^]^ Copyright 2016, American Chemical Society. c) SEM image of welding perovskite NWs. Reproduced with permission.^[^ ^67^ ^]^ Copyright 2020, American Chemical Society. d) SEM image of MWA grown by blade‐coating. Reproduced with permission.^[^ ^35^ ^]^ Copyright 2016, John Wiley and Sons. e) Optical microscopy image of MAPbI_3_ NWs fabricated by roll‐to‐roll micro‐gravure printing technology. Reproduced with permission.^[^ ^75^ ^]^ Copyright 2016, Royal Society of Chemistry. f) SEM image of NWA formed by roller pressing. Reproduced under terms of the CC‐BY license.^[^ ^76^ ^]^ Copyright 2017, Lynn Lee et al., published by Springer Nature. g) SEM image of MAPbI_3_ NWs formed by antisolvent vapor‐assisted crystallization. Reproduced with permission.^[^ ^81^ ^]^ Copyright 2018, American Chemical Society. h) Schematic of the ligand‐assisted reprecipitation process. Reproduced with permission.^[^ ^82^ ^]^ Copyright 2019, American Chemical Society.

Im et al.^[^ ^65^ ^]^ used a two‐step spin‐coating method to grow NWs and fabricated the first perovskite NW solar cell. The perovskite active layer was formed by first spin‐coating DMF solution of PbI_2_, followed by spin‐coating a mixed solution of MAI in IPA and DMF. By controlling the appropriate ratio of DMF, the perovskite structure transitioned from 3D to 1D, resulting in a solar cell with a power conversion efficiency of 14.71% (**Figure** **3**a). Zhu et al.^[^ ^66^ ^]^ first spin‐coated DMF solution of PbCl_2_ and MAI in a 1:3 molar ratio onto a substrate via a one‐step spin‐coating method, followed by annealing to form MAPbI_3_ thin film. They then spin‐coated the mixed solvent of DMF and IPA onto the perovskite film and annealed again. As the solvent evaporated, the perovskite underwent a dissolution–recrystallization process, forming NWs. (Figure 3b). Wu et al.^[^ ^67^ ^]^ employed a two‐step process to form NW networks. Subsequently, a glass slide was placed covered the NWs and the structure was baked in an oven under applied pressure. The overlapping areas of the NW networks underwent secondary growth, achieving a welding‐like effect. The disappearance of the microinterfaces increased the performance, stability, and flexibility of the device (Figure 3c). Further research was conducted on the spin‐coating method for the synthesis of NWs.^[^ ^68^, ^69^, ^70^, ^71^, ^72^, ^73^, ^74^ ^]^

The blade‐coating method's main advantage lies in its ability to fabricate large‐area NWA in a cost‐effective, one‐step process, making it promising for high‐throughput industrial‐scale fabrication. Deng et al.^[^ ^35^ ^]^ reported the preparation of perovskite single‐crystal microwire arrays (MWA) using the simple and low‐cost blade‐coating method. They dragged DMF solution of MAPbI_3_ across a heated plate with a blade. As the blade moved, the small amount of MAPbI_3_ molecules in the solution at the contact line continuously flowed toward the growing sites, self‐assembling and growing under intense intermolecular interactions. The directional movement of the blade controlled the alignment direction of the MWA, and the evaporation of the solution resulted in the formation of MAPbI_3_ MWA (Figure 3d). Hu et al.^[^ ^75^ ^]^ also synthesized MAPbI_3_ NWA by the blade‐coating method and attempted large‐area perovskite NW fabrication using large‐scale roll‐to‐roll micro‐gravure printing technology, but the patterning effect was not good enough (Figure 3e). Lee et al.^[^ ^76^ ^]^ used a roller press method to fabricate NWA, where a cylindrical metal roller is wrapped with a flexible poly(dimethylsiloxane) (PDMS) mold featuring a periodic linear array channel. High‐quality patterned perovskite films were achieved under the optimal rolling speed (Figure 3f). Other reports on the blade‐coating method,^[^ ^77^, ^78^ ^]^ as well as the use of solution shearing,^[^ ^79^ ^]^ have also been documented.

Zhu et al.^[^ ^80^ ^]^ dissolved the MAPbX_3_ (X = I, Br) precursor in polar solvents such as acetonitrile or DMF, and then added it to antisolvents like toluene, which resulted in perovskite crystallizing along a preferred orientation to form NWs. Xu et al.^[^ ^81^ ^]^ dropped MAPbI_3_ droplets under a saturated vapor atmosphere of the antisolvent dichloromethane (DCM). As DCM slowly diffused, it promoted nucleation of MAPbI_3_ NWs at the substrate edges. After the solvent naturally evaporated, NWs were formed at the edges (Figure 3g). The antisolvent vapor atmosphere not only facilitated the crystallization of the perovskite but also effectively prevented moisture from diffusing into the NWs, significantly reducing the formation of surface defects and grain boundaries. Pushkarev et al.^[^ ^82^ ^]^ first sprayed a DMSO precursor solution of CsPbBr_3_ onto a polished and hydrophobized substrate, forming separate droplets. The substrate was then placed in an IPA·H_2_O vapor atmosphere, where IPA acted as an antisolvent to induce precursor precipitation (Figure 3h). The synthesized NWs have been used as hydrogen chloride gas sensors.^[^ ^83^ ^]^ Further studies based on the antisolvent‐assisted crystallization method are also available.^[^ ^84^, ^85^, ^86^, ^87^ ^]^

#### 2.1.2 Colloidal Method

**Figure 4:**
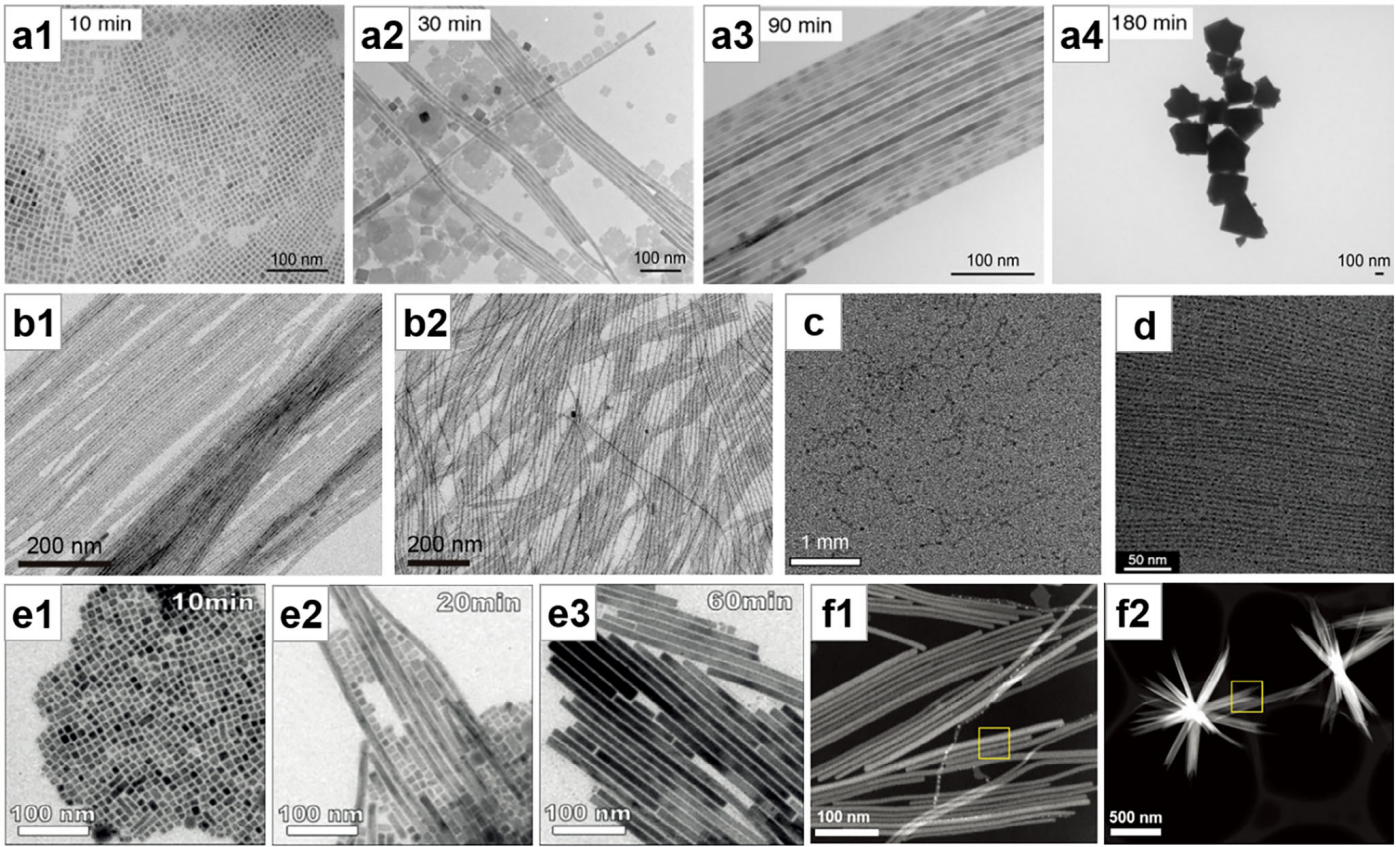
Colloidal synthesis method for NWs growth. a) Typical hot‐injection method: the process of NCs gradually forming NWs and finally disappearing. Reproduced with permission.^[^ ^88^ ^]^ Copyright 2015, American Chemical Society. b) Increasing the ratio of short‐chain carboxylic acid to amine ligands reduces the width of CsPbBr_3_ NWs to b1: 5.1 nm, b2: 3.4 nm. Reproduced with permission.^[^ ^90^ ^]^ Copyright 2016, American Chemical Society. c) “Pearl‐necklace” assemblies intermediates formed during the formation of MAPbBr_3_ NCs into NWs. Reproduced with permission.^[^ ^91^ ^]^ Copyright 2016, American Chemical Society. d) TEM image of high‐purity QWs after stepwise purification. Reproduced with permission.^[^ ^92^ ^]^ Copyright 2016, American Chemical Society. e) TEM images of the transformation process of CsPbBr_3_ NWs synthesized by the ultrasonication‐assisted method. Reproduced with permission.^[^ ^94^ ^]^ Copyright 2017, John Wiley and Sons. f) HAADF‐STEM images of f1: small NW bunches, and f2: large NWs clusters formed by ligand‐assisted reprecipitation process. Reproduced with permission.^[^ ^96^ ^]^ Copyright 2020, John Wiley and Sons.

The solution‐phase growth of NWs can also be achieved through the colloidal method, where NCs are first synthesized and then further converted into NWs, with the hot‐injection method being the most widely used strategy. In this procedure, lead halide is mixed with nonpolar solvent octadecene (ODE), followed by sequential injection of organic ligands (oleylamine and oleic acid (OA)) and cesium oleate at high temperatures, resulting in the formation of CsPbX_3_ NCs.^[^ ^201^ ^]^ The photoluminescence (PL) of the products can be tuned across the entire visible spectrum via anion exchange.^[^ ^202^ ^]^ The synthetic mechanism can be attributed to a seed‐mediated nucleation process, where Pb(0) nanoparticles formed during the reaction act as seeds, providing stable nucleation sites, followed by oriented attachment and self‐assembly growth.^[^ ^203^ ^]^ Building on this synthesis method, Zhang et al.^[^ ^88^ ^]^ extended it to the 1D morphology for the first time. With increasing reaction time, CsPbX_3_ NCs are first generated, followed by the formation of NWs and nanosheets, with NWs eventually becoming the dominant morphology. Over time, these NWs gradually disappear, forming large crystals (**Figure** **4**a). The performance and morphology of NWs can be more precisely tuned by adjusting the type and ratio of ligands.^[^ ^89^, ^204^ ^]^ Through such regulation, thinner NWs have been successfully synthesized, entering the strong quantum confinement regime (Figure 4b,c).^[^ ^90^, ^91^ ^]^ The purity of these QWs can be further improved via a stepwise purification method with the use of antisolvent (Figure 4d).^[^ ^92^, ^93^ ^]^

Tong et al.^[^ ^205^ ^]^ discovered that a simple ultrasonication‐assisted method can be used to fabricate colloidal CsPbX_3_ NCs. In this method, CsPbX_3_ precursors were mixed with the surface‐capping ligands oleylamine and OA in ODE, and then subjected to direct tip sonication to generate CsPbX_3_ NCs. On this basis, as the synthesis time goes by, the morphology changes from NCs to NWs due to the oriented attachment of nanocubes (Figure 4e).^[^ ^94^ ^]^ Sun et al.^[^ ^95^ ^]^ reported a ligand‐assisted reprecipitation method at room temperature, where the precursor solution is dissolved in a good solvent such as DMF, dimethyl sulfoxide (DMSO), or tetrahydrofuran (THF) and then added to a poor solvent such as toluene or hexane at room temperature. In the presence of organic acid and amine ligands, Cs^+^, Pb^2+^, and X^−^ co‐precipitate to form CsPbX_3_. According to the selection of organic acid and amine ligands, the shape of CsPbX_3_ colloidal NCs can be controlled, such as spherical QDs,^[^ ^206^ ^]^ nanocubes, nanorods, and nanoplatelets. Bi et al.^[^ ^96^ ^]^ reported an improved ligand‐assisted reprecipitation process, where halide vacancies and excess amines on the surface of CsPbI_3_ NCs induce anisotropic growth, forming individual NWs. These NWs then undergo vacancy‐driven self‐assembly into clusters (Figure 4f). Numerous other studies have also focused on the colloidal synthesis of NWs.^[^ ^97^, ^98^, ^99^, ^100^, ^101^, ^102^, ^103^, ^104^, ^105^ ^]^

#### 2.1.3 Vapor‐Phase Synthesis

**Figure 5:**
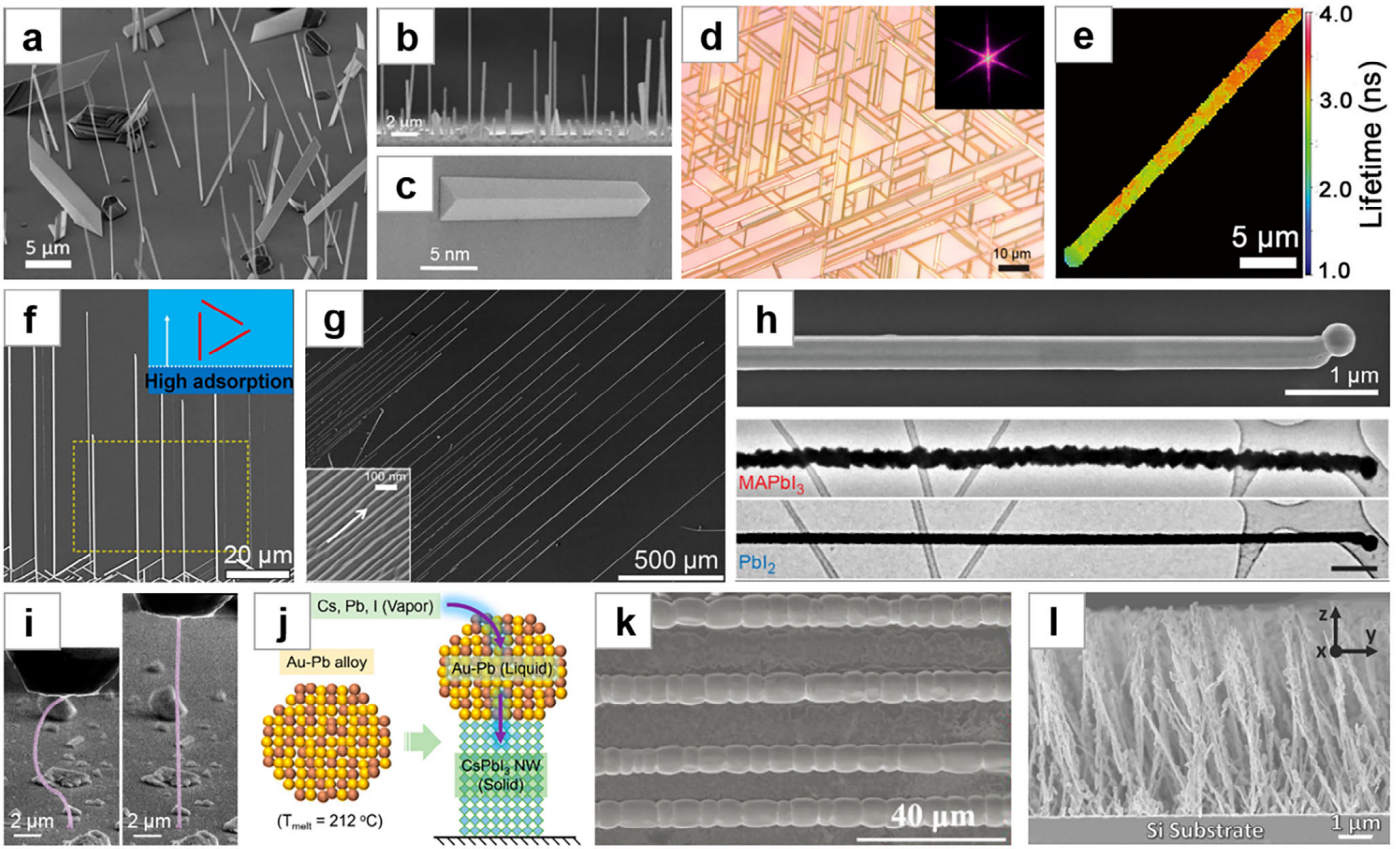
Vapor‐phase synthesis method for NWs Growth. a) SEM image of PbI_2_ NWs grown on Si substrate. Reproduced with permission.^[^ ^106^ ^]^ Copyright 2015, American Chemical Society. b) SEM image of vertically aligned CsPbBr_3_ NWs. Reproduced with permission.^[^ ^107^ ^]^ Copyright 2016, American Chemical Society. c) FIB‐SEM micrograph of a CsPbBr_3_ triangular rod. Reproduced with permission.^[^ ^108^ ^]^ Copyright 2017, American Chemical Society. d) Optical microscopy image of CsPbBr_3_ NW networks grown on phlogopite mica. Reproduced with permission.^[^ ^112^ ^]^ Copyright 2017, American Chemical Society. e) FLIM image of a NW with gradient composition distribution. Reproduced with permission.^[^ ^114^ ^]^ Copyright 2018, John Wiley and Sons. f) SEM image of the angle‐dependent self‐competitive growth results. Reproduced with permission.^[^ ^115^ ^]^ Copyright 2024, American Chemical Society. g) SEM images of the directional CsPbBr_3_ NWs growth along faceted M‐plane sapphire. Reproduced with permission.^[^ ^123^ ^]^ Copyright 2017, American Chemical Society. h) VLS‐grown PbI_2_ NW with Pb catalyst tip and its conversion into MAPbI_3_ NW. Reproduced with permission.^[^ ^131^ ^]^ Copyright 2017, American Chemical Society. i) Reversible elastic deformability of the CsPbBr_3_ NW. Reproduced with permission.^[^ ^133^ ^]^ Copyright 2021, Springer Nature. j) Schematic of the enthalpy‐mediated Au‐seeded growth of CsPbI_3_ NWs. Reproduced with permission.^[^ ^134^ ^]^ Copyright 2023, American Chemical Society. k) SEM image of the CsPbBr_3_ MWA. Reproduced under terms of the CC‐BY‐NC‐ND license.^[^ ^207^ ^]^ Copyright 2020, Yabing Qi et al., published by John Wiley and Sons. l) Cross‐sectional SEM image of glancing‐angle deposition thin films. Reproduced under terms of the CC‐BY‐NC‐ND license.^[^ ^208^ ^]^ Copyright 2022, Juan Ramon Sanchez et al., published by John Wiley and Sons.

Vapor‐phase synthesis methods primarily involve CVD for the epitaxial growth of NWs on substrates such as Si, mica, and sapphire, as well as gas–liquid–solid (GLS) methods. Xing et al.^[^ ^106^ ^]^ employed a two‐step vapor‐phase synthesis to produce MAPbI_3_ NWs. In the first step, PbI_2_ NWs were grown on silicon oxide substrates by CVD method. Both PbI_2_ NWs and bulk crystals grew simultaneously, with the NW morphology being dominant (**Figure** **5**a). The NWs were then transferred to a new substrate using a dry transfer method, followed by the introduction of MAI to convert PbI_2_ into perovskite. Park et al.^[^ ^107^ ^]^ synthesized vertical CsPbX_3_ NWs with a rectangular cross section by directly heating PbX_2_ and CsX powders in a single‐step deposition process (Figure 5b). The end facets of NWs can exhibit various shapes, such as triangular^[^ ^108^ ^]^ (Figure 5c) or square,^[^ ^109^ ^]^ and 2D perovskites like BA_2_PbI_4_ (C_4_H_9_NH_3_PbI_4_) can also be synthesized using similar methods.^[^ ^110^, ^111^ ^]^

Chen et al.^[^ ^112^ ^]^ grew high‐quality CsPbX_3_ NWs networks on mica via vapor‐phase epitaxy. The growth direction of the NWs coincided with the six‐fold symmetry structure present on the mica (001) surface. As the growth time increased, both the NW density and width continued to increase (Figure 5d). This method was also used to synthesize CsSnI_3_ all‐inorganic Sn‐based perovskite NWs on mica.^[^ ^113^ ^]^ Huang et al.^[^ ^114^ ^]^ co‐heated PbI_2_ and PbBr_2_ to epitaxially grow CsPbBr_x_I_3‐x_ NWs on mica. Due to the relatively low evaporation temperature and high vapor pressure of PbI_2_, asynchronous deposition occurred, resulting in a compositional gradient along the NW direction (Figure 5e). Typically, CsPbBr_3_ NWs are epitaxially grown on mica with three symmetric directions due to lattice mismatch limitations, forming network junctions. Fan et al.^[^ ^115^ ^]^ covered graphite sheets on mica to construct a high adsorption area, where NWs oriented perpendicular to the high adsorption area boundaries exhibited a faster growth rate. This preferential growth intercepted NW formation along the other two directions, resulting in NWA aligned perpendicularly to the high adsorption area boundaries (Figure 5f). Many other studies have concentrated on epitaxial growth on mica for the synthesis of NWs.^[^ ^116^, ^117^, ^118^, ^119^, ^120^, ^121^, ^122^ ^]^

Shoaib et al.^[^ ^123^ ^]^ achieved the growth of ultralong NWs through the graphoepitaxial effect on annealed M‐plane sapphire substrates. The CsPbX_3_ precursor was deposited and epitaxially grown at the aligned V‐shaped nanogrooves on the sapphire surface (Figure 5g), leading to the fabrication of high‐performance PDs and lasers.^[^ ^124^, ^125^ ^]^ Oksenberg et al.^[^ ^126^ ^]^ also demonstrated the epitaxial growth of CsPbBr_3_ NWs with different morphologies on M‐ and C‐plane sapphire substrates. As the height of the NWs decreased, the PL emission peak underwent a blue shift, which was the result of bandgap modulation originating from lattice strain caused by heteroepitaxial mismatch.^[^ ^127^ ^]^ Numerous other studies have explored the epitaxial growth of perovskite NWs on sapphire^[^ ^128^, ^129^ ^]^ and other substrates.^[^ ^130^ ^]^

Meyers et al.^[^ ^131^ ^]^ first reported the self‐catalyzed vapor–liquid–solid (VLS) growth of perovskite NWs. Pb was thermally evaporated onto the substrate and melted to form nanoscale Pb droplets, which acted as catalysts during the synthesis process. By heating PbI_2_, which served as the vapor‐phase source of Pb and I, PbI_2_ solid precipitated and continuously grew when the Pb–I equilibrium in the droplets could no longer be maintained, forming high‐quality PbI_2_ NWs with spherical tips. The NWs were then exposed to MAI vapor, leading to in situ conversion into MAPbI_3_ NWs (Figure 5h). Meng et al.^[^ ^132^ ^]^ used nanoscale Sn droplets as catalysts, introducing CsX and PbX_2_ in a 2:1 molar ratio via the gas phase, where supersaturation of liquid Sn induced the direct growth of CsPbX_3_ NWs at the liquid–solid interface. Since no post‐synthetic phase conversion was needed, the resulting crystals exhibited improved quality, the CsPbBr_3_ NWs demonstrated fully reversible elastic deformation (Figure 5i).^[^ ^133^ ^]^ However, residual Sn was found within the NW. Noble metal Au catalyst seeds have also been employed to trigger the VLS growth of CsPbI_3_ NWs.^[^ ^134^ ^]^ Enabled by a spontaneous exothermic nucleation process, the VLS growth temperature was reduced, and the Au‐seeded process would not lead to impurity doping in the perovskite host lattice (Figure 5j).

There are also some other vapor‐phase synthesis methods. Tong et al.^[^ ^207^ ^]^ used the phase transition of the 2D perovskite CsPb_2_Br_5_ to induce the growth of MWA. The deposited CsPb_2_Br_5_ precursor layers underwent high‐temperature annealing and quenching. Due to its layered structure and phase transition, aligned CsBr MWA were obtained without the use of templates. PbBr_2_ thin films were then deposited on it and annealed to obtain CsPbBr_3_ MWA (Figure 5k). Castillo‐Seoane et al.^[^ ^208^ ^]^ initially deposited a highly porous PbI_2_ layer via glancing‐angle vacuum sublimation, followed by the vertical‐angle vapor deposition of MAI, which reacted with the entire structure to form a highly anisotropic MAPbI_3_ nanowall structure, where NWs were formed along the thickness direction (Figure 5l).

#### 2.1.4 Patterning Method

**Figure 6:**
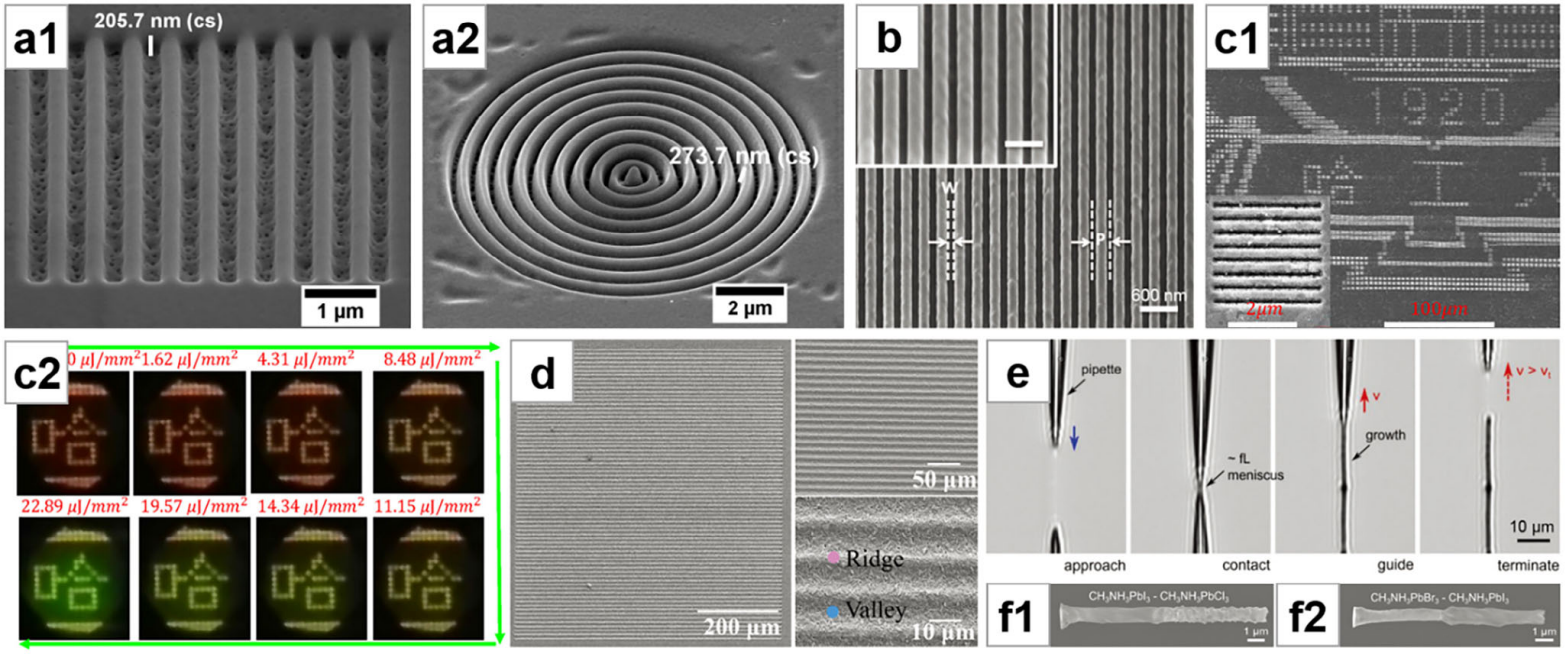
Direct Patterning of Perovskites to Form NWs. a) SEM images of a1: grating a2: circular structure by FIB etching. Reproduced under terms of the CC‐BY license.^[^ ^135^ ^]^ Copyright 2015, Alias, Mohd Sharizal et al., published by American Vacuum Society. b) SEM image of nanograting fabricated from MAPbI_3_ thin film. Reproduced with permission.^[^ ^138^ ^]^ Copyright 2017, John Wiley and Sons. c) c1: SEM image of patterned grating structure. c2: Microscope images of dynamically adjustable color display at different pumping densities. Reproduced with permission.^[^ ^139^ ^]^ Copyright 2018, American Chemical Society. d) Grating array patterned on FAPbI_3_ film by femtosecond DLW. Reproduced with permission.^[^ ^140^ ^]^ Copyright 2022, John Wiley and Sons. e) Optical microscopy images of the 3D nano‐printing process of perovskite NWs. Reproduced with permission.^[^ ^141^ ^]^ Copyright 2019, John Wiley and Sons. f) 3D Nano‐printing FE‐SEM images of f1: MAPbI_3_‐MAPbCl_3_, f2: MAPbBr_3_‐MAPbI_3_ NW HJT. Reproduced under terms of the CC‐BY‐NC license.^[^ ^142^ ^]^ Copyright 2023, Mojun Chen et al., published by John Wiley and Sons.

Direct patterning of perovskites has also been reported as a method for fabricating perovskite NWs, including techniques such as focused ion beam (FIB) etching, electron beam lithography (EBL), direct laser writing (DLW), and 3D nano‐printing. Alias et al.^[^ ^135^ ^]^ first reported a patterning strategy for MAPbBr_3_ crystals using FIB etching, achieving nanoscale precision (>63 nm) to pattern the perovskite crystals, creating linear or circular grating structures (**Figure** **6**a). However, due to surface damage caused by Ga ion bombardment, the PL intensity decreased by ≈60%. A subsequent report^[^ ^136^ ^]^ introduced a method using gas‐assisted FIB etching, where gases such as XeF_2_ and I_2_ were introduced during the ion milling process, enhancing etching efficiency and reducing surface damage. However, ion implantation and surface amorphization induced by FIB technology remain unavoidable sources of damage.^[^ ^137^ ^]^ Gholipour et al.^[^ ^138^ ^]^ used FIB to etch patterned nanograting and nanoslits on perovskite films, studying the effects of grating period and milling depth on light absorption and emission (Figure 6b). Gao et al.^[^ ^139^ ^]^ patterned MAPbBr_3_ thin films using EBL followed by inductively coupled plasma etching with chlorine gas, producing a patterned grating structure. Different grating periods resulted in various external structural colors, and varying laser intensities could control the brightness of the perovskite PL emission. The color tuning of PL emission, corresponding to the external structural colors, allowed for dynamic and tunable color displays by adjusting laser intensity on specific grating structures (Figure 6c). Tian et al.^[^ ^140^ ^]^ used femtosecond DLW to create grating arrays on FAPbI_3_ perovskite films. As the grating period decreased, the peaks and valleys of the grating exhibited a triangular sawtooth pattern (Figure 6d). Due to the low reflectivity and high absorption of the triangular micro‐grating, the structure exhibited excellent emission and detection performance.

Chen et al.^[^ ^141^ ^]^ developed a simple and versatile 3D nano‐printing method for organic–inorganic metal halide perovskites. A nanopipette filled with perovskite ink was first brought into contact with a Si substrate, forming a meniscus at the contact point. The pipette was then moved at a micrometer‐scale speed, and as the solvent evaporated, crystals grew continuously in the direction of movement to form NWs. The crystal growth could be terminated by suddenly increasing the pulling speed beyond the threshold value (Figure 6e). This method was further applied to 3D print perovskite NW HJT.^[^ ^142^ ^]^ Using a double‐barrel nanopipette as a printing nozzle, the tip filled with MAPbBr_3_ precursor was first brought into contact with the Si substrate to grow MAPbBr_3_ NW. Next, the tip with MAPbI_3_ precursor was used to contact the MAPbBr_3_ NW, forming MAPbBr_3_‐MAPbI_3_ NW HJT (Figure 6f). Though the NWs produced by this method had lower crystal quality and were difficult to fabricate in high throughput, the approach allowed for free design of NW shapes and the fabrication of various materials for HJT. Other methods, such as inkjet printing,^[^ ^143^ ^]^ have also been explored.

### 2.2 Template‐Assisted Methods

The morphology of perovskites can be controlled by using different templates. There are two main approaches when growing NWs by template‐assisted methods. One is film confinement, where a perovskite film is first formed, and then a template is used to imprint and confine the growth of NWs, the other is template‐guided growth, where perovskite is driven into the pre‐formed template, aligning according to the style of the template. There are numerous mechanisms that can be utilized in this process, and the final result is often the outcome of the induction of multiple factors. Similarly, vapor phase synthesis of NWs can also be carried out with the assistance of templates. Regularly aligned NWA, due to fewer junctions and shorter carrier transport paths, typically exhibit higher photoresponsivity. Compared to randomly distributed NWs, they also offer better orientation, uniformity, and reproducibility—resulting in enhanced polarization sensitivity, flexibility, and image sensing capabilities, while also being more favorable for integration.

#### 2.2.1 Film Confinement

The film confinement method uses a template to cover a pre‐formed perovskite film for spatial confinement, allowing perovskite NWs to form after crystallization. The imprinting method is widely applicable to various materials, has low requirements for wettability, and offers unique advantages in forming complex isolated patterns. However, residual material may remain between patterned structures. It is also worth noting that due to limited solubility and viscosity, perovskite films fabricated by solution‐based methods can only achieve a thickness of ≈700 nm.^[^ ^140^ ^]^

**Figure 7:**
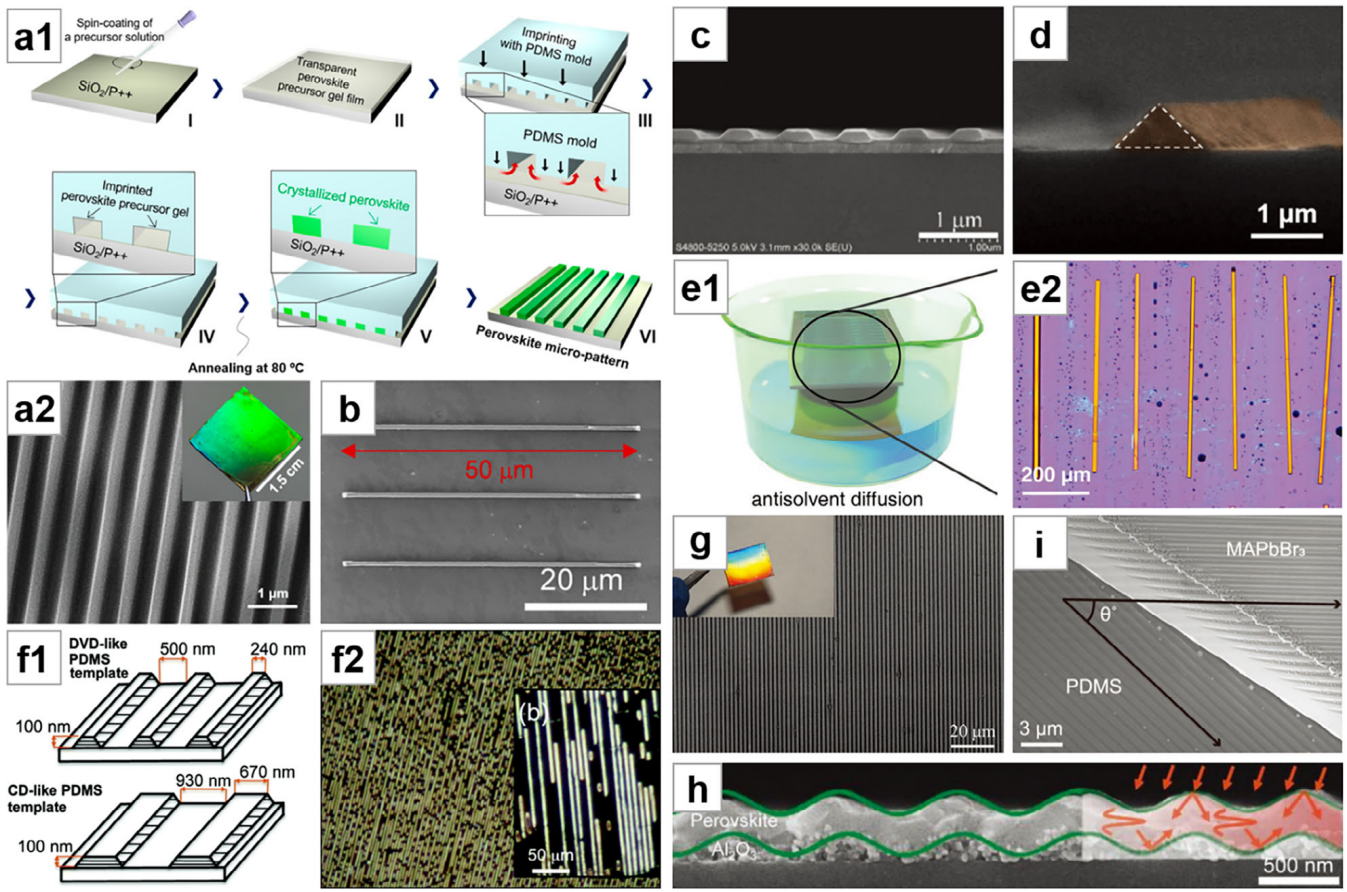
Film Confinement Method for NWs Growth. a) a1: Schematic illustration of the solvent‐assisted gel printing procedure. a2: FE‐SEM image of MAPbBr_3_ NWA. Reproduced with permission.^[^ ^144^ ^]^ Copyright 2016, American Chemical Society. b) SEM image of MAPbBr_3_ NWA. Reproduced with permission.^[^ ^145^ ^]^ Copyright 2017, American Chemical Society. c) Cross‐sectional SEM image of MAPbI_3_ NWA. Reproduced with permission.^[^ ^146^ ^]^ Copyright 2017, John Wiley and Sons. d) Side‐view SEM image of the single crystal MAPbI_3_ Triangular NWA. Reproduced with permission.^[^ ^147^ ^]^ Copyright 2023, John Wiley and Sons. e) e1:Schematic of the template‐confined antisolvent crystallization device. e2: Optical image of aligned CsPbBr_3_ MWA. Reproduced with permission.^[^ ^148^ ^]^ Copyright 2019, John Wiley and Sons. f) f1:The structure of CD and DVD disc original master. f2: The optical images of the CsPbBr_3_ NWA obtained on DVD and CD replica PDMS substrates. Reproduced with permission.^[^ ^149^ ^]^ Copyright 2017, Royal Society of Chemistry. g) SEM image of the CsPbI_3_ NWA and the interference phenomenon of the film. Reproduced with permission.^[^ ^150^ ^]^ Copyright 2020, John Wiley and Sons. h) Cross‐sectional SEM image of perovskite Moiré lattices structures. Reproduced with permission.^[^ ^151^ ^]^ Copyright 2021, John Wiley and Sons. i) SEM image of the moiré perovskite. Reproduced with permission.^[^ ^152^ ^]^ Copyright 2022, John Wiley and Sons.

PDMS is an excellent template transfer material. Before curing, it exhibits outstanding plasticity and adhesiveness, while after curing, it possesses good chemical stability, shear resistance, and hydrophobicity.^[^ ^209^ ^]^ When coated onto a NW hard template, PDMS can fill the recessed nanostructures, enabling high‐precision replication of morphology and dimensions. The resulting soft template after curing offers excellent stability and accuracy. Moreover, Li et al. found that the removal of the PDMS template caused minimal damage to the perovskite NWs.^[^ ^152^ ^]^ Jeong et al.^[^ ^144^ ^]^ used PDMS transfer‐printed patterned Si as a template and spin‐coated perovskite solution with DMSO as the solvent onto the substrate. Due to the slow evaporation rate of DMSO at room temperature, the unannealed film has a soft‐gel state. When pressed with the PDMS template, the film has sufficient fluidity to enter the pores of the pattern, and then anneal to form NWA (**Figure** **7**a). Liu et al.^[^ ^145^ ^]^ employed a similar approach by placing a PDMS template onto the drop‐coating perovskite solution and applying slight pressure, which also led to the formation of NWA (Figure 7b). Mao et al.^[^ ^146^ ^]^ covered the solid MAPbI_3_ thin film with a PDMS template and exposed it to a dry gaseous CH_3_NH_2_ (MA) atmosphere, causing the MAPbI_3_ to convert into transparent liquid intermediate CH_3_NH_3_PbI_3_⋅CH_3_NH_2_. The film was subsequently annealed again to form MAPbI_3_ NWA (Figure 7c). Xiong et al.^[^ ^147^ ^]^ performed photolithography and anisotropic etching on Si to fabricate a triangular NWA hard template. Then they used poly(methyl methacrylate) (PMMA) and PDMS for a concave–convex–concave transfer process to obtain PDMS soft templates. The NWA synthesized via the confined growth method exhibited triangular morphology, which further increased the surface area and provided a more robust structure, benefiting both photoresponse and mechanical flexibility (Figure 7d). Yang et al.^[^ ^148^ ^]^ used DLW and etching to fabricate 100‐µm‐high silicon micropillars and obtained templates via PDMS transfer printing. The DMSO solution of CsPbBr_3_ precursor was pre‐saturated with acetonitrile (CH_3_CN) and then dropped onto the substrate. After placing the PDMS template, pressure and vacuum treatment were applied, followed by growth in a closed atmosphere with CH_3_CN, where crystallization was assisted by the antisolvent, ultimately yielding high‐quality MWA (Figure 7e).

Optical discs are easily accessible and can be used as materials for the original master of NWs. The pre‐etched grooves on CD and DVD discs are spirally distributed from the center, which can be regarded as parallel 1D structures at the microscale.^[^ ^149^, ^210^ ^]^ However, it is evident that relying on optical discs as the master template limits the diversity of the NWs. Rodriguez et al.^[^ ^149^ ^]^ obtained the PDMS mold of optical discs and directly spin‐coated the perovskite precursor solution onto the wettability‐treated PDMS surface. Upon annealing, NWA were formed, although their continuity was not particularly ideal (Figure 7f). Wang et al.^[^ ^150^ ^]^ used PDMS transfer from CD discs as NW templates, spin‐coated CsPbI_3_ precursor solution on the substrate, and then pressed the PDMS template onto the film, followed by vacuum treatment and gradient temperature annealing. Their study found that a precursor concentration of 0.6 M was required to produce clear and high‐quality NWA (Figure 7g). Moiré lattices are formed by superimposing two identical periodic structures with relative rotation angles.^[^ ^211^ ^]^ Song et al.^[^ ^151^ ^]^ used PDMS to replicate DVD discs pattern and first imprinted it onto a spin‐coated Al_2_O_3_ film to obtain a grating‐structured substrate. MAPbI_3_ was then spin‐coated onto the substrate, followed by a second nanoimprinting step to fabricate a perovskite layer with a top–bottom‐grating (Moiré lattice) structure (Figure 7h). This design enhanced the device's light collection capability due to the synergistic effects of reduced reflection and increased absorption. Li et al.^[^ ^152^ ^]^ created a Moiré lattice structure by dripping perovskite between two PDMS templates prepared in the same way. They first deposited MAPbBr_3_ on PDMS template 1, then covered it with another PDMS template 2. The grating directions of the two layers of PDMS had an angle θ, and due to the confinement effect of the two nanograting structure templates, the top and bottom surfaces of the perovskite NWs exhibited grating structures with a relative rotation angle θ (Figure 7i). There are many other studies using film confinement methods to fabricate NWA.^[^ ^153^, ^154^, ^155^, ^156^, ^157^ ^]^

#### 2.2.2 Template Guidance

The general idea of the template‐guided method is to first form a patterned template and then introduce the perovskite solution into the template. Some methods follow the template‐free approach, such as spin‐coating and blade‐coating, but the shape is standardized by the microchannel template. Others utilize capillary force, wettability, and other driving forces.

**Figure 8:**
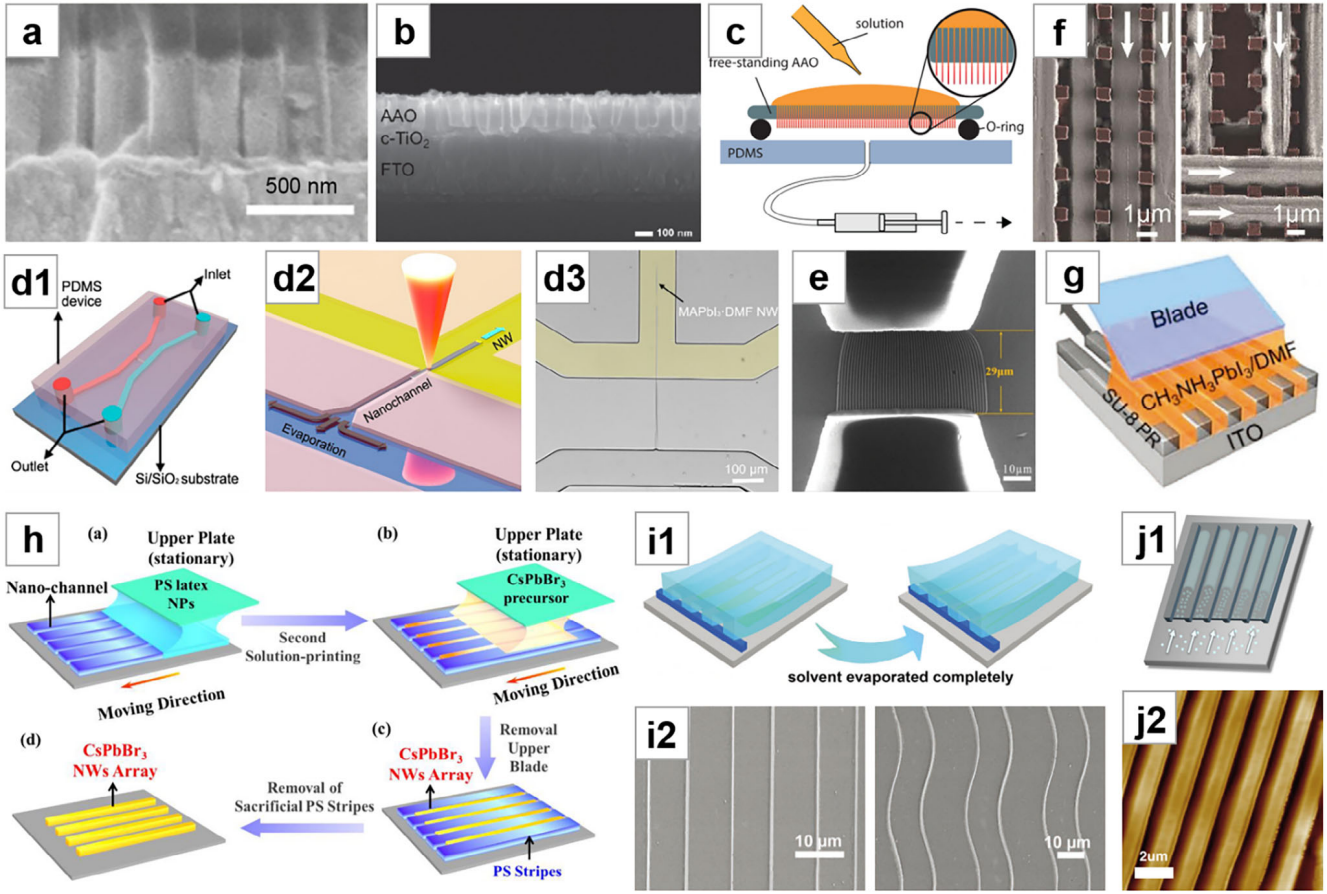
Template‐guided method for NWs growth (I). a) Cross‐sectional SEM image of NWA synthesized by AAO template. Reproduced with permission.^[^ ^158^ ^]^ Copyright 2016, American Chemical Society. b) NWA synthesized by AAO template as an absorption layer. Reproduced with permission.^[^ ^159^ ^]^ Copyright 2016, John Wiley and Sons. c) Perovskite NW extrusion scheme. Reproduced under terms of the CC‐BY‐NC‐ND license.^[^ ^162^ ^]^ Copyright 2017, American Chemical Society. d) d1: Schematic of the microfluidic device. d2: Growth mechanism of NW. d3: Real‐time monitoring of NW growth. Reproduced with permission.^[^ ^166^ ^]^ Copyright 2018, American Chemical Society. e) SEM image of the silicon NW microfluidic templates. Reproduced with permission.^[^ ^167^ ^]^ Copyright 2020, Royal Society of Chemistry. f) Guiding effect in non‐continuous‐walled channels. Reproduced under terms of the CC‐BY license.^[^ ^168^ ^]^ Copyright 2016, Massimo Spina et al., published by Springer Nature. g) Schematic illustration of the blade coating of NWA. Reproduced with permission.^[^ ^170^ ^]^ Copyright 2020, John Wiley and Sons. h) Schematic of the capillary‐assisted solution printing method. Reproduced with permission.^[^ ^171^ ^]^ Copyright 2020, John Wiley and Sons. i) i1:Schematic of the flow of perovskite precursor in the microchannel. i2: SEM images of straight and curved MAPbBr_3_ MWA. Reproduced with permission.^[^ ^173^ ^]^ Copyright 2020, John Wiley and Sons. j) j1:Schematic of infiltration of capillary channels by capillary condensation. j2: AFM image of MAPbI_3_ NWA. Reproduced under terms of the CC‐BY license.^[^ ^175^ ^]^ Copyright 2025, Gangjian Hu et al., published by Springer Nature.

Porous Anodic Aluminum Oxide (AAO) and the two‐step anodization process, since their introduction,^[^ ^212^ ^]^ have been widely used for the synthesis of various nanomaterials.^[^ ^213^ ^]^ This sophisticated technique enables the fabrication of vertically aligned NWA. Ashley et al.^[^ ^158^ ^]^ were the first to use AAO for the synthesis of perovskite NWs. First, they evaporated aluminum onto the substrate and performed anodization to induce nanopore formation. The distribution and pore size of the pores can be controlled by adjusting the applied anodization voltage and the pore refinement step. Subsequently, spin‐coating was employed to drive the perovskite into the nanopores under the combined action of capillary and centrifugal forces, leading to the formation of NWA (**Figure** **8**a). Kwon et al.^[^ ^159^ ^]^ facilitated the infiltration of the precursor solution into the porous AAO membrane by combining vacuum and spin‐coating methods (Figure 8b), where the concentration of the precursor solution and the spin speed had a significant impact on the filling level of the pores.^[^ ^214^ ^]^ Similar results were also achieved by first drop‐coating the perovskite, then covering it with the AAO template, followed by spin‐coating^[^ ^160^ ^]^ or mild thermal treatment,^[^ ^161^ ^]^ demonstrating the role of capillary force in guiding the perovskite into the template. Oener et al.^[^ ^162^ ^]^ created a sealed chamber by placing an O‐ring between the AAO template and PDMS membrane. With the perovskite dropped onto the AAO template, they evacuated the air from the chamber, creating a pressure difference. Under the driving pressure gradient, the perovskite solution quickly filled the AAO pores (Figure 8c). Lin et al.^[^ ^163^, ^164^ ^]^ also used a vacuum to guide perovskite, but applied inkjet printing to deposit the perovskite. Kwon et al.^[^ ^165^ ^]^ reported a sol–gel‐derived solution route that exploited the excellent infiltration properties of PbAc_2_ solution to fill the pores, which was subsequently converted stepwise into PbO, PbI_2_, and MAPbI_3_.

Zhou et al.^[^ ^166^ ^]^ employed a microfluidic device developed by their group.^[^ ^215^ ^]^ The PDMS template contains two microchannels connected by a nanochannel. By injecting perovskite solution into one of the microchannels and monitoring the growth of perovskite NWs in real time, the mechanism of crystallization growth was revealed (Figure 8d). Chen et al.^[^ ^167^ ^]^ also employed microfluidic templates to fabricate perovskite NWA (Figure 8e).

Spina et al.^[^ ^168^ ^]^ fabricated a channel array on Si through patterned photoresist and then dripped the supersaturated MAPbI_3_ solution in DMF at one end of the channel. Driven by capillary force, the solution flowed along the channels and formed NWA after the photoresist was removed. Notably, even in non‐continuous‐walled channels, the guiding effect was still observed (Figure 8f). They combined the perovskite NWA with graphene to fabricate a vertical composite device, achieving a responsivity of up to 6 × 10^6^ A W^−1^, which is the highest value reported for perovskite NWs. Lim et al.^[^ ^169^ ^]^ extended the spin‐coating approach by spin‐coating perovskite onto stripe‐patterned PMMA templates, followed by annealing to obtain NWA. Deng et al.^[^ ^170^ ^]^ synthesized MAPbI_3_ NWA via the blade‐coating method, where the perovskite solution was loaded into the region between the photoresist stripe array and the blade. Due to capillary effect, the solution filled the microchannel, and by adjusting the coating speed to match the crystallization speed, high‐quality NWs with an ultralow defect density of 2 × 10^9^ cm^−3^ and long carrier lifetime of 175 ns were achieved (Figure 8g). Pan et al.^[^ ^171^ ^]^ reported a capillary‐assisted solution printing method. According to previous reports,^[^ ^216^ ^]^ blade‐coating a water‐diluted polystyrene latex particle suspension can generate periodic nanochannel‐like cracks. Based on this structure, the perovskite precursor solution was subsequently blade‐coated, and driven by capillary forces, the perovskite infiltrated the microchannels and self‐assembled into NWA (Figure 8h).

Li et al.^[^ ^172^ ^]^ transferred patterned Si template by PDMS and bonded it onto the substrate. After dropping precursor solution onto one end of the template, the solution was absorbed and filled the microchannels under the guidance of capillary force, leading to the formation of (BA)_2_(MA)_n‐1_Pb_n_Br_3n+1_ 2D perovskite MWA. Subsequently, Li et al.^[^ ^173^ ^]^ optimized the process by silanizing the photolithographed Si template with hydrophobic trichloro(1H,1H,2H,2H‐perfluorooctyl)silane (FOTS) before PDMS transfer and attachment to the substrate. A spacer was used on one side of the template to form an opening, and MAPbBr_3_ precursor was deposited on the opposite side. Driven by capillary force and solvent vapor escape upon heating, the perovskite solution entered and flowed along the microchannels. Preferential crystallization occurred along the microchannel sidewalls due to surface adsorption, resulting in the growth of two MWs along both sidewalls of each channel. Meanwhile, FOTS molecules were also transferred via PDMS onto the MW surfaces, forming an in situ encapsulation layer (Figure 8i). HJT could also be fabricated by depositing different perovskite precursor solutions on each side of the template.^[^ ^174^ ^]^ However, when relying solely on capillary forces, NW growth becomes limited as the filling distance approaches the submicron scale.^[^ ^217^ ^]^ To overcome this, Hu et al.^[^ ^175^ ^]^ developed a method based on capillary condensation^[^ ^218^ ^]^ to drive NW growth. A PDMS template replicated from CDs was laminated onto a substrate and placed at an angle inside a sealed quartz crucible. Upon addition of the perovskite precursor solution and subsequent heating, the combined effects of capillary force and capillary condensation enabled the solution to fill the nanochannels. After annealing, high‐quality, centimeter‐long, independent perovskite NWA were formed (Figure 8j).

**Figure 9:**
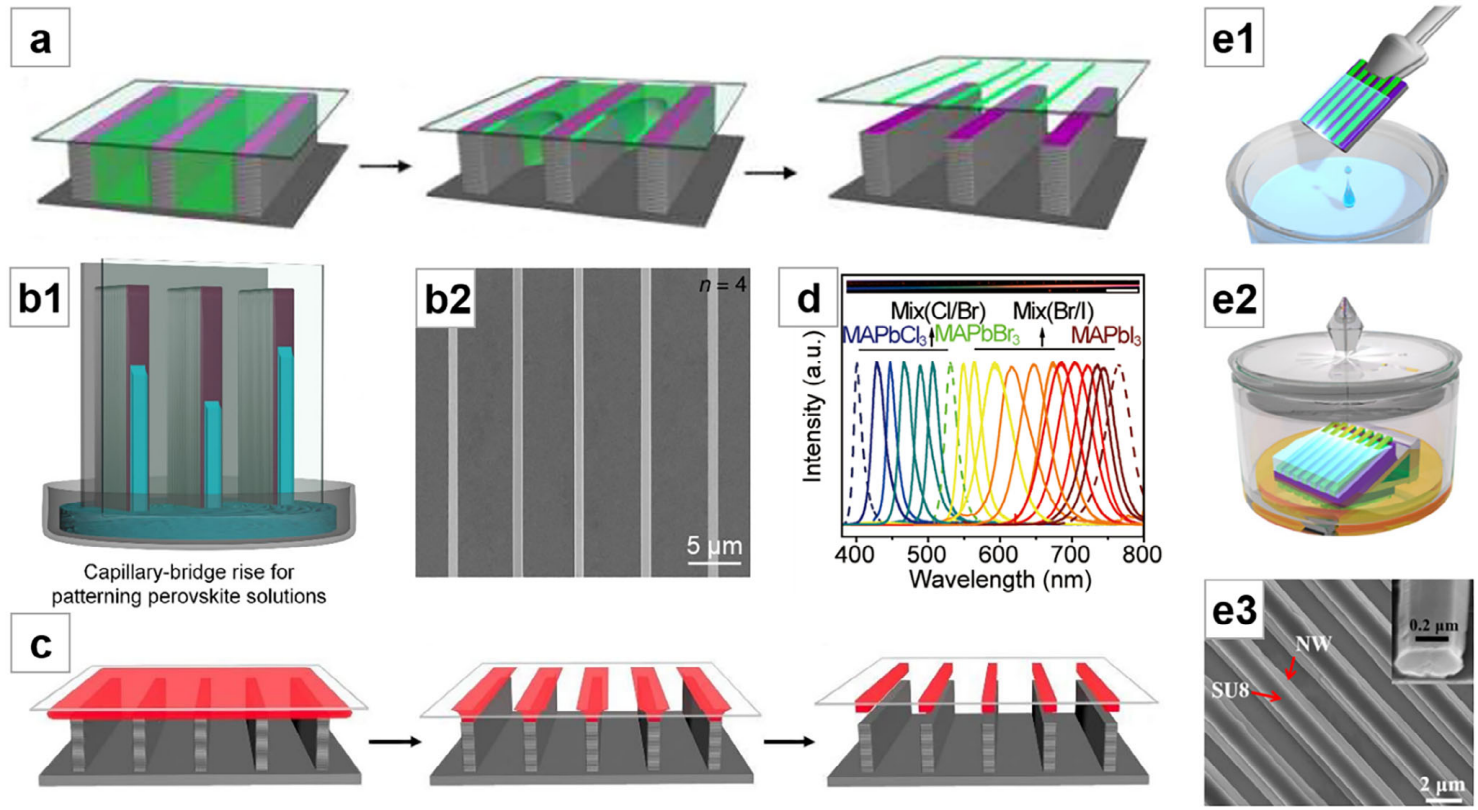
Template‐guided method for NWs growth (II). a) Schematic of NWA growth through lyophilic top and lyophobic sidewalls template. Reproduced with permission.^[^ ^176^ ^]^ Copyright 2017, John Wiley and Sons. b) b1: Schematic of NWA growth through lyophobic top and lyophilic sidewalls template. b2: SEM image of high quality (BA)_2_(MA)_n‐1_Pb_n_I_3n+1_ (n = 4) NWA. Reproduced with permission.^[^ ^32^ ^]^ Copyright 2018, Jiangang Feng et al., published by Springer Nature. c) Schematic of the improved asymmetric‐wettability template‐guided method. Reproduced with permission.^[^ ^177^ ^]^ Copyright 2019, John Wiley and Sons. d) Fluorescence micrograph of the typical compositionally graded MAPbX_3_ MW and the corresponding in situ fluorescence emission spectrum. Reproduced with permission.^[^ ^39^ ^]^ Copyright 2023, John Wiley and Sons. e) e1,e2: Schematic of fluid‐guided antisolvent vapor‐assisted crystallization method for the fabrication of MAPbI_3_ NWA. e3: SEM image of MAPbI_3_ NWA grown on the two sides of SU‐8 photoresist strips. Reproduced with permission.^[^ ^184^ ^]^ Copyright 2017, American Chemical Society.

The wettability of the substrate can also effectively guide the perovskite solution. Feng et al. prepared a topographical template via photolithography and then created asymmetric‐wettability topographical templates in two ways. The first method^[^ ^176^ ^]^ involved modifying the top of the template's micro‐pillars with the hydrophobic molecule heptadecafluorodecyltrimethoxysilane (FAS). After dropping the perovskite precursor solution onto the template and covering the Si substrate, the solution was confined within the gaps between the micro‐pillars. As the solvent evaporated, capillary trailing occurred at the lyophobic–lyophilic boundary, leading to nucleation and growth of perovskite, and the formation of a CsPbBr_3_ NWA on the target substrate (**Figure** **9**a). The second method^[^ ^32^ ^]^ involved protecting the top of the template's micro‐pillars with photoresist, then modifying the sidewalls and bottoms with FAS. After removing the photoresist, the sidewalls became lyophobic while the top remained lyophilic. The template was then vertically immersed in the perovskite precursor solution. Under the combined effects of capillary force and Laplace pressure, the perovskite solution rose along the lyophilic path, resulting in the formation of 2D perovskite single‐crystal NWA of (BA)_2_(MA)_n‐1_Pb_n_I_3n+1_. The NWA exhibited strict alignment, precise position, and homogeneous size (Figure 9b). Chen et al.^[^ ^177^ ^]^ from the same group followed the approach of treating the sidewalls with hydrophobic molecules, then dropped the CsPbI_3_ precursor solution onto the prepared template, covering it with Si substrate to assist in forming a liquid film. As the mild annealing solvent evaporated, stable α‐CsPbI_3_ NWA were formed under the regulation of Laplace pressure and capillary force (Figure 9c). Fu et al.^[^ ^39^ ^]^ used a similar method to fabricate MAPbBr_3_ MWA, and then slowly inserted both ends of the arrays into Oleylammonium chlorine (OAmCl) and Oleylammonium iodide (OAmI) solutions in ODE through stepper motor control for transformation. This process achieved a gradual MAPbCl_3_‐MAPbBr_3_‐MAPbI_3_ structure within a single MW (Figure 9d). The detectivity of the devices all exceeded 10^15^ Jones, with MAPbI_3_ reaching an astonishing 9.77 × 10^15^ Jones. This improved method was also used to synthesize α‐FAPbI_3_ NWA,^[^ ^178^ ^]^ tunable‐bandgap MAPbX_3_ NWA,^[^ ^179^ ^]^ (ThMA)_2_(MA)_n‐1_Pb_n_I3_n+1_ 2D perovskite NWA,^[^ ^38^ ^]^ and (R‐/S‐α‐PEA)_2_PbI_4_ chiral 2D perovskite NWA,^[^ ^36^ ^]^ etc., and more extended research on asymmetric‐wettability was done.^[^ ^180^, ^181^, ^182^, ^183^ ^]^

Deng et al.^[^ ^184^ ^]^ employed the fluid‐guided antisolvent vapor‐assisted crystallization method to grow perovskite NWA. First, they patterned photoresist on a Si substrate via photolithography and immersed the photoresist template in MAPbI_3_ solution. Then, they dragged the template out along with the hanging solution and placed it on an inclined glass surface inside a sealed bottle. The bottle contained DCM antisolvent, whose saturated vapor gradually diffused into the perovskite solution, causing the MAPbI_3_ to crystallize. Due to the difference in wettability between the photoresist and substrate, the perovskite grew directionally along the sidewalls of the photoresist stripes (Figure 9e). Kashtiban et al.^[^ ^219^ ^]^ ground and mixed oxidized single‐walled carbon nanotubes (SWCNTs) with diameters ranging from 1.2 to 1.7 nm with CsPbBr_3_ or CsSnI_3_, respectively, followed by high‐temperature heating. The molten CsPbBr_3_ and CsSnI_3_ infiltrated the interior of the SWCNTs, forming the smallest isolated perovskite NW structures.

#### 2.2.3 Template‐Assisted Vapor‐Phase Synthesis

Template‐assisted vapor‐phase synthesis is based on vapor‐phase deposition methods, where the template controls the morphology of the perovskite. Many studies have utilized porous AAO membranes as templates.

**Figure 10:**
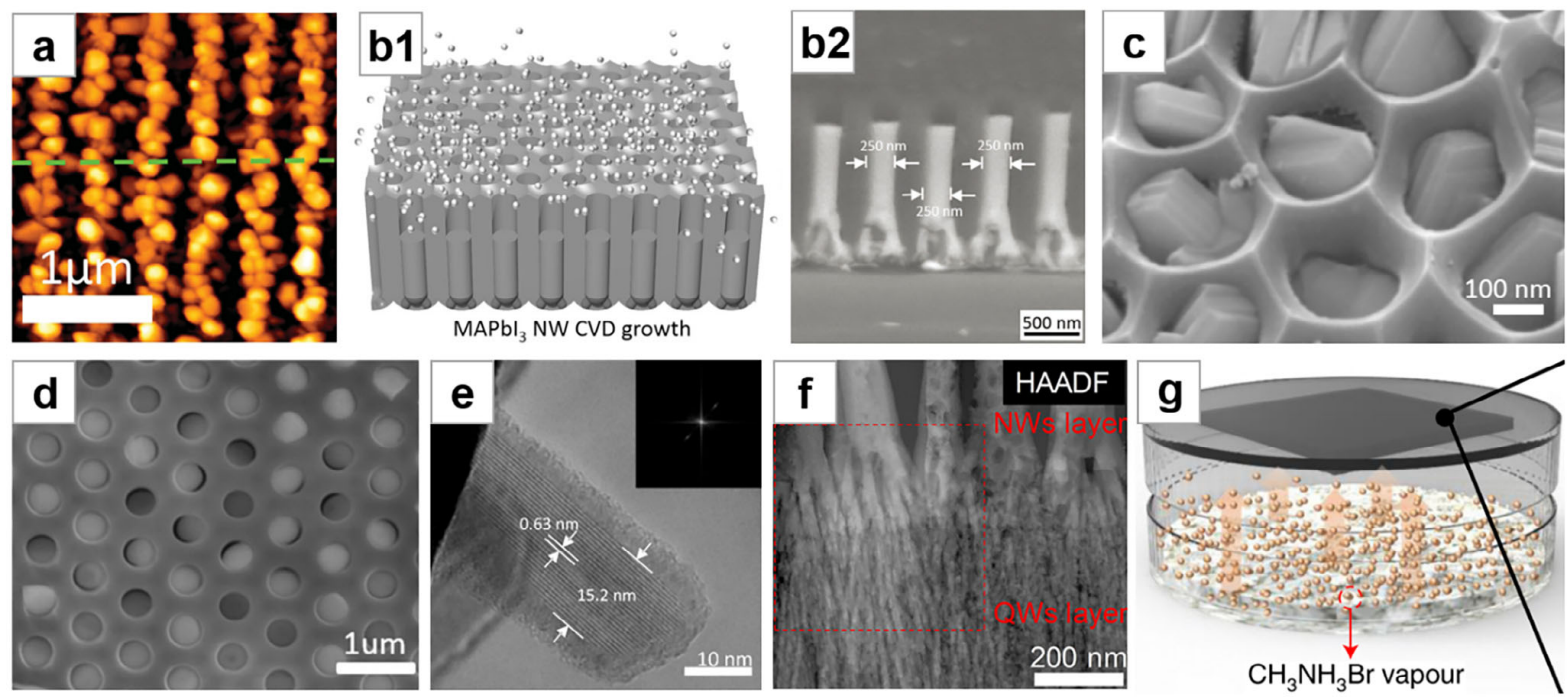
Template‐assisted vapor‐phase synthesis method for NWs growth. a) AFM image after the perovskite is evaporated on the polymer resist. Reproduced with permission.^[^ ^185^ ^]^ Copyright 2015, John Wiley and Sons. b) b1: Schematic of the VSSR process for growing NWA using porous AAO membrane as a template. b2: Cross‐section view of MAPbI_3_ NWA in AAO after growth. Reproduced with permission.^[^ ^186^ ^]^ Copyright 2016, John Wiley and Sons. c) Top SEM image of CsPbI_3_ NWA inside AAO. Reproduced with permission.^[^ ^189^ ^]^ Copyright 2017, American Chemical Society. d) SEM image of MASnI_3_ NWA inside AAO. Reproduced with permission.^[^ ^190^ ^]^ Copyright 2016, American Chemical Society. e) HRTEM image of MAPbI_3_ QWA. Reproduced with permission.^[^ ^191^ ^]^ Copyright 2019, American Chemical Society. f) HAADF‐STEM image of the MAPbI_3_ QW/NW junction. Reproduced with permission.^[^ ^192^ ^]^ Copyright 2022, American Chemical Society. g) Schematic of the CSVR setup. Reproduced with permission.^[^ ^193^ ^]^ Copyright 2022, Daquan Zhang et al., published by Springer Nature.

After the dual‐source thermal evaporation system for perovskite gas‐phase deposition was reported,^[^ ^220^ ^]^ Saliba et al.^[^ ^185^ ^]^ simultaneously deposited PbCl_2_ and MAI onto the substrate via the dual‐source evaporation system, where the evaporated perovskite conformally coated onto the underlying corrugated polymer resist, resulting in a glass/corrugated polymer/perovskite stack structure (**Figure** **10**a).

Gu et al.^[^ ^186^ ^]^ first reported a vapor–solid–solid reaction (VSSR) method for growing perovskite NWs using a porous AAO membrane as a template, where MAI vapor reacts with Pb nanoclusters at the bottom of vertical nanochannels. The AAO was prepared by imprinting aluminum foil with a dense pillar‐array mold, followed by two‐step anodization and etching. A layer of Pb was then electrochemically deposited at the bottom of the AAO channels. MAI vapor was introduced and reacted with Pb nanoclusters. Pb was partially converted into an intermediate product PbI_2_, and ultimately fully transformed into MAPbI_3_ NWA (Figure 10b). Each individual vertical NW could serve as a pixel, and the fabricated device was a photodiode‐type detector. Many extended studies have been conducted based on this method.^[^ ^187^, ^188^ ^]^ Waleed et al.^[^ ^189^ ^]^ synthesized all‐inorganic CsPbI_3_ NWA by reacting CsI vapor with Pb nanoclusters (Figure 10c). They also electrochemically deposited Sn nanoclusters at the bottom of the nanopores of AAO template and reacted them with MAI vapor to synthesize lead‐free MASnI_3_ NWA (Figure 10d).^[^ ^190^ ^]^ Zhang et al.^[^ ^191^ ^]^ obtained quantum wire arrays (QWA) with an average pore size of 5.7 nm by controlling the anodization voltage, and the quantum confinement effect and nanophotonic effect improved the PLQY (Figure 10e). As the pore size of the AAO template is directly proportional to the anodization voltage, Zhang et al.^[^ ^192^ ^]^ first obtained large‐pore AAO template by anodizing at high voltage, followed by a barrier thinning process and then anodized at low voltage to achieve ultrafine pores. They then used VSSR to synthesize a unique NW‐QW junction (Figure 10f). Zhang et al.^[^ ^193^ ^]^ further improved the process by inverting the Pb‐deposited AAO template and placing it above a container filled with MABr powder, and then performing a close‐spaced vapor reaction (CSVR) to grow the QWs by overall baking. The MAPbBr_3_ QWs exhibited a PLQY of 92%, and the emission spectrum could be tuned by adjusting the halide source. The average diameter of the QWA can be precisely reduced through the atomic layer deposition Al_2_O_3_ conformal coating process.^[^ ^194^ ^]^ Due to the vertical growth of the NWA, the devices are fabricated as longitudinal structures, which enable various unique applications, such as solar cells,^[^ ^195^ ^]^ resistive random‐access memory (Re‐RAM),^[^ ^196^, ^197^, ^198^ ^]^ spherical LEDs,^[^ ^193^ ^]^ and biomimetic eyes.^[^ ^22^, ^199^ ^]^

## 3 Modification of Nanowires

Lead‐based or tin‐based perovskite NWs grown through general synthesis methods, whether all‐inorganic or organic–inorganic hybrid perovskite, often have potential for performance improvement if not specially treated. This chapter introduces several modification methods to enhance the performance or achieve special properties of perovskite NWs, such as using special perovskite materials like 2D layered perovskites, chiral perovskites, and perovskite materials that are not tin‐based or lead‐based perovskites, constructing perovskite HJT structures, incorporating special materials or encapsulating agents to improve performance and stability.

### 3.1 Perovskite Material Selection

2D perovskites are formed by inserting organic long chains into 3D perovskite structures, with the general composition A_m_’A_n‐1_B_n_X_3n+1_​, where A’ represents the organic long chain, A is the organic cation, and *n* inorganic layers of [BX_6_] sandwich *m* layers of A’ ligands to form a 2D layered structure. Depending on the number of inorganic layers *n*, the material is classified as pure 2D when n = 1, and quasi‐2D when 1 < n ≤ 5.^[^ ^221^ ^]^ For 3D lead halide perovskites, they are generally regarded as non‐exciton materials,^[^ ^222^ ^]^ which can achieve efficient free charge generation. However, in 2D or quasi‐2D perovskites, as the thickness of the inorganic layers *n* decreases, the quantum confinement effect becomes stronger, leading to an increased exciton binding energy, exhibiting strong excitonic properties.^[^ ^223^ ^]^ This results in a blue shift of both absorption and emission peaks,^[^ ^224^ ^]^ as well as a widening of the bandgap.^[^ ^225^ ^]^ The number of layers *n* can be controlled to some extent by adjusting precursor composition and controlling the temperature and duration of the synthesis process.^[^ ^226^ ^]^

**Figure 11:**
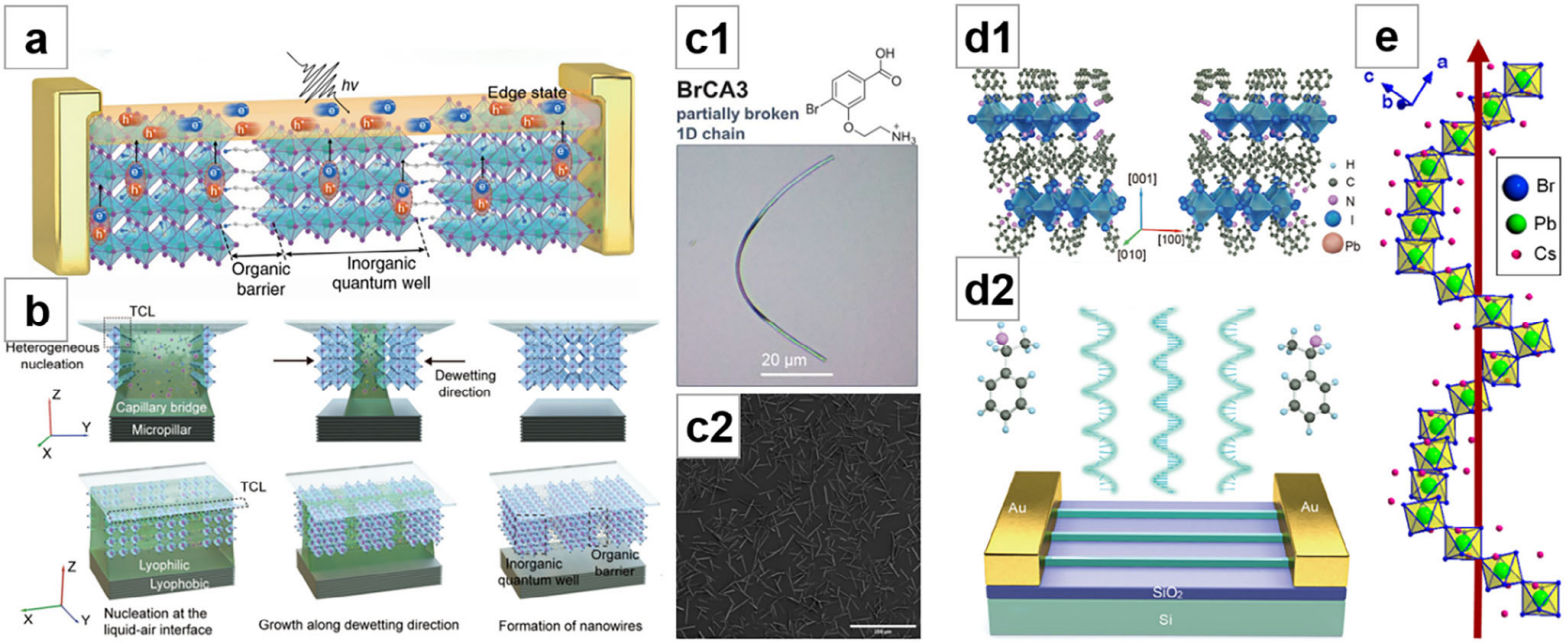
Perovskite Material Selection. a) Scheme of carrier dynamics in the PD of single‐crystalline (101)‐oriented 2D perovskite. Reproduced with permission.^[^ ^32^ ^]^ Copyright 2018, Jiangang Feng et al., published by Springer Nature. b) Schematic of the crystallization of layer‐perovskite NWs in a capillary bridge. Reproduced with permission.^[^ ^38^ ^]^ Copyright 2020, John Wiley and Sons. c) c1: BrCA3 molecule and the morphology of the NW. c2: Morphology of NWs of (BrCA3)_2_PbBr_4_. Reproduced with permission.^[^ ^228^ ^]^ Copyright 2024, Wenhao Shao et al., published by The American Association for the Advancement of Science. d) d1: Crystal structures of (S‐α‐PEA)_2_PbI_4_ (left) and (R‐α‐PEA)_2_PbI_4_ (right) chiral 2D‐perovskite NWs. d2: Schematic illustration of Stokes‐parameter PD. Reproduced with permission.^[^ ^36^ ^]^ Copyright 2021, American Chemical Society. e) Schematic of the spiral rotation of the octahedra along the long axis of the NWs. Reproduced with permission.^[^ ^102^ ^]^ Copyright 2024, American Chemical Society.

Blancon et al.^[^ ^227^ ^]^ discovered that in 2D perovskites with n > 2, localized low‐energy electronic states form at the edges of the layers. These edge states provide a direct path for exciton dissociation into free charge carriers. Additionally, when carriers are trapped in these edge states, they are protected from energy loss through non‐radiative processes. Feng et al.^[^ ^32^ ^]^ mentioned that in 2D perovskite NWs, there exist serial hopping barriers in the charge transport path within the crystal, which effectively suppress dark current. Due to the narrow width of the NWs, excitons can efficiently diffuse to the crystal edges and dissociate into long‐lived free carriers. The high photoconductive channels provided by the exposed crystal edges enable a significant enhancement in photocurrent. This structure, with high resistance in the crystal interior and high conductivity at the crystal edges, achieves a responsivity greater than 1.5 × 10^4^ A W^−1^ and a detectivity exceeding 7 × 10^15^ Jones, demonstrating ultra‐sensitive photodetection (**Figure** **11**a). Expanding on this, Zhao et al.^[^ ^38^ ^]^ characterized the ordered nucleation and unidirectional growth along the dewetting direction during the growth process, using an improved asymmetric‐wettability template‐guided method (Figure 11b). This advancement further increased the detectivity to over 9.1 × 10^15^ Jones. Li et al.^[^ ^172^ ^]^ synthesized (BA)_2_(MA)_n‐1_Pb_n_Br_3n+1_​ NWA through a template‐assisted method, which also exhibited an ultrahigh detectivity exceeding 1 × 10^15^ Jones. Ghoshal et al.^[^ ^110^ ^]^ synthesized (BA)_2_PbI_4_​ NWs via the vapor deposition method and demonstrated excellent PL emission polarization and polarization detection performance. Similar work was also reported by Yadav et al.^[^ ^111^ ^]^ Kamminga et al.^[^ ^154^ ^]^ synthesized (C_6_H_5_CH_2_NH_3_)_2_PbI_4_ ((PMA)_2_PbI_4_) 2D perovskite MWA using both imprinting thin films and capillary force‐guided methods. Zhao et al.^[^ ^72^ ^]^ obtained (BA)_2_Cs_n‐1_Pb_n_Br_3n+1_ quasi‐2D perovskite NWs through a simple spin‐coating process, achieving dual‐pulse laser emission under single‐pulse femtosecond laser excitation. Shao et al.^[^ ^228^ ^]^ reported a molecular templating method, synthesizing molecules such as TPA3 and BrCA3, which restricted crystal growth along all crystallographic directions except for^[^ ^110^ ^]^ and promoted 1D growth. This approach led to the formation of 2D layered perovskite NWs, including (BrCA3)_2_PbI_4_, (BrCA3)_2_SnI_4_, (BrCA3)_2_MAPb_2_Br_7_, and (BrCA3)_2_MA_2_Pb_3_I_10_. Compared to phenylethylamine (PEA)‐based structures, these candidates exhibited enhanced stability against ambience, heat, and light (Figure 11c).

Chirality refers to the property of an object that cannot be superimposed onto its mirror image. Due to their unique micro‐helical structure, chiral perovskites have emerged as promising building blocks for circularly polarized light (CPL) detection. Zhao et al.^[^ ^36^ ^]^ synthesized α‐PEAI by dissolving α‐PEA in HI, then adding it to the solution of PbO in HI and H_3_PO_2_. This precursor was then processed to form perovskite precursors, which were successively synthesized into (S‐α‐PEA)_2_PbI_4_, (R‐α‐PEA)_2_PbI_4_, and (rac‐α‐PEA)_2_PbI_4_ chiral perovskite NWA with left‐handed, right‐handed, and racemic properties, respectively, through the asymmetric wettability template‐guided method. Due to the addition of chiral molecules, the response of the (R‐α‐PEA)_2_PbI_4_ and (S‐α‐PEA)_2_PbI_4_ NW devices exhibited significant differences under left‐handed circularly polarized light (LCP) and right‐handed circularly polarized light (RCP) illumination (Figure 11d). Liu et al.^[^ ^181^ ^]^ employed (R)‐(+)‐𝛼‐methylbenzylamine (R‐MBA) or (S)‐(‐)‐𝛼‐methylbenzylamine (S‐MBA) as chiral ligands, mixing them with (CH_3_COO)_2_Pb and HI to form the precursor, and similarly synthesized (R‐/S‐MBA)_2_PbI_4_ chiral perovskite NWA, achieving CPL detection. Wang et al.^[^ ^156^ ^]^ selected two isomers of the chiral ligand naphthyl ethylamine (NEA) to synthesize (R/S‐NEA)PbI_3_ chiral perovskites. The Stokes detector was constructed by using the advantages of thin film and NW morphology distribution for circular polarization and linear polarization detection respectively. Guha et al.^[^ ^102^ ^]^ reported the construction of chirality in all‐inorganic perovskite NWs without chiral ligands. They first synthesized CsCdBr_3_ NWs via the colloidal method and then chiral CsPbBr_3_ NWs were fabricated by B‐site cation exchange. The intrinsic chirality originated from the lattice rotation. The CsPbBr_3_ NWs were not simple 1D straight chains but were formed by corner‐connected NCs. When the NCs connected, slight relative twists occurred between adjacent cubes, resulting in the overall chirality of the structure (Figure 11e). As the NWs grew longer, the overall chirality intensified, leading to CPL emission.

Some perovskite materials, which are not tin‐based or lead‐based perovskites, have also been used. Li et al.^[^ ^87^ ^]^ synthesized CsCu_2_I_3_ NWs by titrating anhydrous acetonitrile solutions of CsI and CuI with antisolvent ether, and obtained a specific photoresponse in the ultraviolet band of 230 to 350 nm, which is not achievable by common lead‐based and tin‐based perovskite NWs. The resulting UV PDs demonstrated excellent detection performance, polarization sensitivity, and flexibility. Poddar et al.^[^ ^198^ ^]^ utilized an AAO template to electrodeposit Bi clusters, and then introduced MAI vapor to generate MA_3_Bi_2_I_9_ NWA using VSSR, which can be used for Re‐RAM.

### 3.2 Construction of Heterojunctions

**Figure 12:**
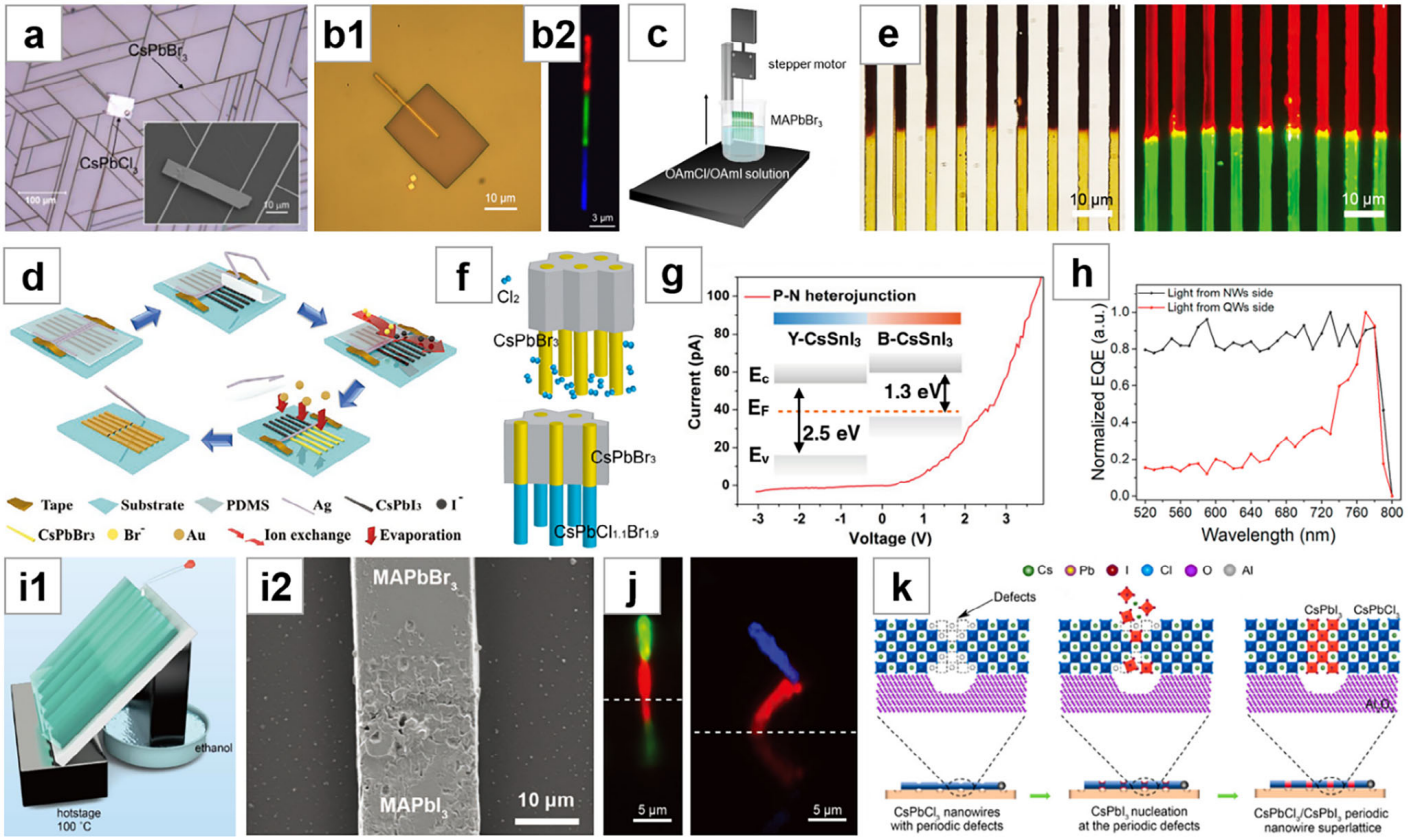
Perovskite NW HJT. a) Optical image and SEM image of a representative heterostructure of a CsPbCl_3_ microplate placed over CsPbBr_3_ NW networks. Reproduced with permission.^[^ ^119^ ^]^ Copyright 2018, American Chemical Society. b) b1: Optical microscope image of a CsPbBr_3_ NW partially coated with PMMA. b2: Confocal PL mapping of a three‐color NW HJT. Reproduced under terms of the CC‐BY‐NC‐ND license.^[^ ^56^ ^]^ Copyright 2017, Peidong Yang et al., published by National Academy of Sciences. c) Schematic of the device used for liquid anion exchange via dipping. Reproduced with permission.^[^ ^39^ ^]^ Copyright 2023, John Wiley and Sons. d) Schematic of the HJT fabrication process. Reproduced with permission.^[^ ^150^ ^]^ Copyright 2020, John Wiley and Sons. e) Microscopy image and fluorescence microscopy image of a typical p–n junction NWA. Reproduced with permission.^[^ ^182^ ^]^ Copyright 2022, John Wiley and Sons. f) Schematic of the anion exchange process. Reproduced under terms of the CC‐BY license.^[^ ^161^ ^]^ Copyright 2022, Zhaojun Zhang et al., published by American Chemical Society. g) I–V characteristics of the p–n HJT. Reproduced under terms of the CC‐BY‐NC‐ND license.^[^ ^59^ ^]^ Copyright 2018, Yang et al., published by National Academy of Sciences. h) EQE spectra with light from both the NW and the QW sides. Reproduced with permission.^[^ ^192^ ^]^ Copyright 2022, American Chemical Society. i) i1: Schematic of temperature gradient holding system. i2: SEM image of the HJT structure. Reproduced with permission.^[^ ^174^ ^]^ Copyright 2022, John Wiley and Sons. j) Straight two‐color MAPbI_3_‐MAPbBr_3_ wire and MAPbI_3_‐MAPbCl_3_ zigzag through 3D printing. Reproduced under terms of the CC‐BY‐NC license.^[^ ^142^ ^]^ Copyright 2023, Mojun Chen et al., published by John Wiley and Sons. k) side view cross‐sectional growth model of the CsPbCl_3_/CsPbI_3_ periodic NW superlattices. Reproduced with permission.^[^ ^231^ ^]^ Copyright 2024, American Chemical Society.

HJT refers to solid‐state interfaces between two different materials. The fabrication of perovskite HJT can exhibit unique functionalities that are unattainable from individual components, such as rectification effects, self‐powered detection, and narrowband photodetection. Pan et al.^[^ ^119^ ^]^ directly transferred dip‐coated perovskite microplates onto epitaxially grown CsPbBr_3_ NWs on mica, observing the anion interdiffusion process, which naturally formed HJT with gradient halide compositions (**Figure** **12**a). More studies have been reported on the fabrication of NW HJT based on solid–solid anion exchange, which can be obtained by cross‐contact annealing.^[^ ^84^, ^85^, ^120^ ^]^ Sun et al.^[^ ^121^ ^]^ sputtered a layer of amorphous indium–gallium–zinc oxide (IGZO) onto the surface of CsPbBr_3_ NWA epitaxially grown on mica, forming HJT that enhanced light absorption in the violet spectrum.

Anion exchange in both liquid and vapor phases is also commonly employed for the fabrication of HJT. Dou et al.^[^ ^56^ ^]^ transferred CsPbBr_3_ NWs grown by dip‐coating through a micromanipulator, then partially covered the NW with PMMA via EBL. Subsequently, the structure was immersed in OAmX (X = Cl, I) solution for anion exchange, resulting in two‐ and three‐color HJT structures (Figure 12b). Xu et al.^[^ ^229^ ^]^ synthesized single MAPbBr_3_ MW via simple space‐limit crystallization and then immersed it in IPA solution of MAI and MACl using the pull method via dipping, resulting in a compositionally graded MAPbX_3_ MW with practical spectral recognition and colorful imaging capabilities. Fu et al.^[^ ^39^ ^]^ used an asymmetric‐wettability template to fabricate MAPbBr_3_ MWA, and then slowly and gradually inserted the two ends into the ODE solution of OAmCl and OAmI respectively for transformation under stepper motor control, thereby obtaining a gradient bandgap‐adjustable perovskite MWA (Figure 12c). The device has excellent detection performance and can be used for accurate color recognition.

Wang et al.^[^ ^150^ ^]^ blade‐coated a layer of PDMS on CsPbI_3_ NWA grown by the template confinement method. After peeling off half of the PDMS, they employed a vapor‐phase ion exchange process to in situ convert CsPbI_3_ into CsPbBr_3_, thereby forming a CsPbI_3_‐CsPbBr_3_ lateral HJT PD (Figure 12d). Guan et al.^[^ ^182^ ^]^ adopted a similar method, covering a selective area of MAPbBr_3_ NWA with PMMA film and gradually replaced Br by HI gas, yielding MAPbBr_3_/MAPbBr_3‐x_I_x_ p‐n junction structures (Figure 12e). Liu et al.^[^ ^230^ ^]^ also adopted a similar idea to fabricate a CsPbBr_3_/CsPb(Br_1‐x_Cl_x_)_3_ HJT. Zhang et al.^[^ ^161^ ^]^ placed an AAO template above the CsPbBr_3_ solution. After mild heating, NWs grew in the AAO pores, with some extending from the bottom of the template. During the subsequent vapor‐phase anion exchange process in Cl_2_ environment, the NWs inside the AAO were protected, while the extended NWs outside the template were converted into halide perovskite with mixed chlorine–bromine anions, resulting in the formation of HJT (Figure 12f).

Kong et al.^[^ ^59^ ^]^ found that during the phase transition of CsSnI_3_ NWs induced by heating, a switch in electrical properties from n‐ to p‐type occurs. By locally inducing partial phase transitions through heating, they formed p–n junctions that exhibited pronounced current rectification behavior (Figure 12g). Zhang et al.^[^ ^192^ ^]^ fabricated dual‐diameter AAO templates through a two‐step electrochemical anodization process and synthesized NW‐QW junctions using the VSSR method. Owing to the excellent carrier transport capability of the NW layer and the wider bandgap and higher carrier recombination rate of the QW layer, when illuminated from the QW side, only photons with energies between the bandgaps of the QW and NW layers can pass through the QW and be absorbed and collected by the NW. This results in a unique narrowband photodetection effect (Figure 12h). Li et al.^[^ ^174^ ^]^ first added MAPbI_3_ solution at one end of a substrate covered with a PDMS template to grow MWs, then brought this end into contact with an annealing source while physically cooling the other end. MAPbBr_3_ solution was introduced from the cooled side, allowing the MAPbBr_3_ precursor to nucleate and grow on the pre‐formed MAPbI_3_ MWs. This process ultimately formed MAPbI_3_‐MAPbBr_3_ MW HJT (Figure 12i). Owing to the presence of the built‐in electric field, the transport of photogenerated electrons and holes was significantly enhanced, enabling the device to achieve excellent detection performance even under self‐powered conditions. Chen et al.^[^ ^142^ ^]^ fabricated a standalone perovskite NW HJT through 3D printing, allowing for precise control over the shape and composition of the NW HJT (Figure 12j). Lv et al.^[^ ^231^ ^]^ used photolithography to create a periodic pore array as nucleation sites for HJT formation. M‐plane sapphire α‐Al_2_O_3_ was annealed at 1700 °C to form “line‐shaped grooves.” CsPbCl_3_ NWs grew horizontally along these grooves via VLS mechanism under the guidance of the catalyst and formed fracture defects at the pore positions. Cl^−^ ions could be precisely exchanged and completely replaced with I^−^ ions at the periodic fracture defects, eventually forming 1D periodic CsPbCl_3_/CsPbI_3_ HJT and obtaining a superlattice structure (Figure 12k).

### 3.3 Additives, Passivation, and Encapsulation

Stability remains the greatest challenge for perovskite materials. Halide perovskites are essentially salts, and without proper encapsulation, they readily absorb moisture. In addition, high temperatures can also break bonds and disrupt the crystal structure. Prolonged exposure to environmental factors such as moisture, oxygen, and heat leads to degradation, significantly compromising their optoelectronic performance, shortening device lifetimes, and ultimately hindering the commercialization of perovskite technologies.^[^ ^232^, ^233^ ^]^ Stability can be effectively improved by defect passivation through additives or by encapsulation to block moisture and oxygen. Moreover, incorporating certain functional materials into perovskites can further enhance or extend their performance.^[^ ^234^ ^]^

**Figure 13:**
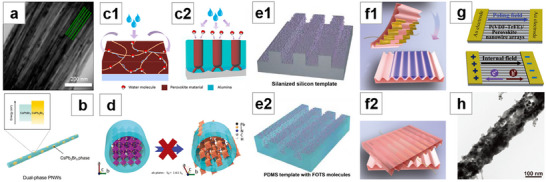
Additives, passivation, and encapsulation. a) After insertion into a polymer the NWs retain their overall size and structure. Reproduced with permission.^[^ ^97^ ^]^ Copyright 2016, American Chemical Society. b) Schematic of the synthesis of dual‐phase NWs. Reproduced with permission.^[^ ^105^ ^]^ Copyright 2022, John Wiley and Sons. c) c1: Schematic of tin perovskite thin film decay from moisture. c2: Tin perovskite NWs sample schematic demonstrating sidewall protection of AAO from moisture. Reproduced with permission.^[^ ^190^ ^]^ Copyright 2016, American Chemical Society. d) Schematic of the mechanism of the AAO prohibiting the α‐to‐δ phase transition of FAPbI_3_ NWs through spatial confinement. Reproduced with permission.^[^ ^187^ ^]^ Copyright 2018, Royal Society of Chemistry. e) e1: Schematic of silicon template with a layer of FOTS molecules on the surface after silylation. e2: Schematic of the PDMS template with silane molecular layer. Reproduced with permission.^[^ ^173^ ^]^ Copyright 2020, John Wiley and Sons. f) Schematic of in situ encapsulated Moiré lattice perovskite PDs. Reproduced with permission.^[^ ^152^ ^]^ Copyright 2022, John Wiley and Sons. g) Schematic of the ferroelectric polarization‐induced formation of internal electric field in the NWA device. Reproduced with permission.^[^ ^153^ ^]^ Copyright 2019, John Wiley and Sons. h) TEM image of an individual UCNP–perovskite composite. Reproduced with permission.^[^ ^63^ ^]^ Copyright 2018, John Wiley and Sons.

Deng et al.^[^ ^42^ ^]^ demonstrated the use of a spin‐coated hydrophobic polymer PMMA to cover the NWs, coupled with a poly(ethylene terephthalate) (PET) substrate, which not only blocked water vapor and improved the interface contact quality, but also passivated defects, which helped to improve stability. In the case of colloidally synthesized NWs, Raja et al.^[^ ^97^ ^]^ dispersed the NWs in polymer matrices such as poly(styrene–ethylene–butylene–styrene) (SEBS) through toluene (**Figure** **13**a). The stability of the NWs was significantly improved through macroscale polymer encapsulation, and the composite material retained a high PLQY even after being immersed in water for four months. This improvement is likely attributed to the matched interface, which minimizes ligand loss during the encapsulation process. Shin et al.^[^ ^105^ ^]^ reported a dual‐phase passivation strategy, in which colloidally synthesized CsPbBr_3_ NWs were reacted in a PbBr_2_‐rich atmosphere, leading to a localized phase transition from CsPbBr_3_ to CsPb_2_Br_5_. The CsPb_2_Br_5_ phase served as a passivation layer for CsPbBr_3_, effectively reducing the trap density (Figure 13b). Waleed et al.^[^ ^190^ ^]^ used porous AAO templates to fabricate MASnI_3_ Sn‐based perovskite NWs. The AAO templates offered excellent protection, significantly inhibiting the lateral diffusion of water and oxygen molecules (Figure 13c). The spatial confinement provided by the AAO templates can also prevented lattice distortion and volume expansion of the perovskite NWs, ultimately forming a stable cubic phase (Figure 13d).^[^ ^187^, ^189^ ^]^ As a result, the photocurrent decay in the device was 500 times slower than that of thin film devices. Further improvement in stability was achieved by applying an epoxy passivation layer on the NW device. Li et al.^[^ ^173^ ^]^ evaporated a layer of hydrophobic FOTS molecules onto a patterned Si template for silylation. They then transferred the pattern from the Si template onto a PDMS stamp. During this transfer, some of the FOTS molecules partially adhered to the PDMS surface. The perovskite MWs guided by this template also have a layer of FOTS molecules on their surface, forming an in situ encapsulation layer (Figure 13e). After being exposed to air for 342 days, the device maintained 96% of its initial photocurrent. PDMS can not only serves as a template material but also functions as an excellent encapsulation material. Li et al.^[^ ^152^ ^]^ further utilized two layers of PDMS with a relative rotation angle θ to confine the fabrication of perovskite NW grating structures. The upper PDMS layer contained electrodes and wires, making PDMS the in situ encapsulation layer for the device (Figure 13f). Due to the super hydrophobicity of PDMS, the stability of the in situ encapsulated device was significantly enhanced, with performance retaining 95.7% after 3 h of exposure to water vapor, and 95% after 223 days in air.

Gao et al.^[^ ^44^ ^]^ explored three NW passivation strategies: oxygen plasma exposure, MAI IPA soaking, and OA toluene soaking. Since OA can passivate the exposed Pb ions on the surface of MAPbI_3_ NWs through a deprotonation process, it forms lead‐oleate bond (Pb─OOC─R). The resulting hydrophobic oleate sheath also encapsulates the MAPbI_3_ NWs. The devices after OA passivation exhibited the best performance, reducing the dark current while increasing the photocurrent, obtaining lower trap density, higher carrier mobility, and longer carrier lifetime. Furthermore, the environmental stability of the devices was also enhanced. Xia et al.^[^ ^70^ ^]^ introduced a small amount of chlorobenzene solution of the organic semiconductor dioctylbenzothieno [2,3‐b] benzothiophene (C8BTBT) into the perovskite, and spin‐coated to synthesize a MAPbI_3_/C8BTBT bulk HJT. The C8BTBT layer served as an efficient hole extraction layer to enhance hole transport due to its intrinsic high mobility and energy level matching, while also acting as a waterproof layer to prevent perovskite degradation. Asuo et al.^[^ ^71^ ^]^ demonstrated that directly incorporating lead thiocyanate (Pb(SCN)_2_) into the precursor solution facilitated the synthesis of stable mixed perovskite NW networks under ambient conditions. This stability was attributed to the enhanced chemical bonding in the lattice due to solid ionic interactions between SCN^−^ and adjacent Pb and the hydrogen bond between SCN^−^ and MA^+^. The authors also verified that increasing the concentration of PbI_2_ precursor could passivate the boundaries between perovskite grains, improving the performance of perovskite‐based PDs. When the PbI_2_ concentration was increased from 0.2 M to 0.8 M, the photocurrent increased by 15 times. Chen et al.^[^ ^177^ ^]^ doped polyvinylpyrrolidone (PVP) into the CsPbI_3_ perovskite precursor and synthesized NWA through an asymmetric‐wettability template. They found that NWs with 10 wt% PVP exhibited the longest PL lifetime and the highest PL intensity. The addition of PVP plays a key role in the long‐term stability of the α‐phase CsPbI_3_ NWs. Chen et al.^[^ ^178^ ^]^ then introduced 10 mol% of PEA^+^ into the FAPbI_3_ perovskite precursor, using the same method to synthesize NWA. The content of PEA^+^ was not high and was not incorporated into the FAPbI_3_ lattice. Quasi‐2D perovskites were not generated, but the formation of stable α‐FAPbI_3_ perovskites was promoted. Cao et al.^[^ ^153^ ^]^ incorporated the organic ferroelectric material poly(vinylidene‐fluoride‐trifluoroethylene) (P(VDF‐TrFE)) into the perovskite precursor and fabricated composite NWA on polyethylene naphthalate (PEN) substrates using the imprinting method. The ferroelectric semiconductor can be polarized by an external electric field, generating an internal electric field that separates electron–hole pairs. The P(VDF‐TrFE)/perovskite hybrid NWA enhanced the strength of the ferroelectric field in perovskite, providing a stronger built‐in field that facilitates the separation and transport of photogenerated carriers, and realizes self‐power supply (Figure 13g). Wu et al.^[^ ^73^ ^]^ incorporated the ionic liquid 1‐butyl‐3‐methylimidazoliumtetrafluoroborate (BMIMBF_4_) as an additive into MAPbI_3_ NWs. The introduction of BMIMBF_4_ not only passivated defects in the MAPbI_3_ NWs to inhibit degradation but also adsorbed onto the NW surface, forming rapid charge transport channels to enhance charge transport. Unencapsulated devices showed no performance degradation even after being exposed to air for over 5000 h. Chang et al.^[^ ^69^ ^]^ spin‐coated an n‐type conjugated polymer, poly[2,5‐bis(2‐dodecylhexadecyl)‐3,6‐di(thiophen‐2‐yl)pyrrolo‐[3,4‐c]pyrrole‐1,4(2H,5H)‐dione‐alt‐(E)‐1,2‐bis(3‐cyanothiophen‐2‐yl)ethene] (DPP‐CNTVT), onto the fabricated perovskite NW network as a multi‐functional interfacial layer. The Lewis base‐rich functional groups in DPP‐CNTVT effectively passivated under‐coordinated Pb^2+^ defects by forming Lewis adducts, thereby enhancing the stability and optoelectronic performance of the perovskite NWs. Furthermore, by adopting polymer barrier films comprising UV‐curable epoxy/fluoropolymer‐coated PEN layer as the encapsulation layer, devices with long‐term stability of up to 15300 h were achieved. Rare earth‐doped upconversion nanoparticles (UCNPs) represented by NaYF_4_:Ln (Ln = Yb/Er, Er) have the unique ability to continuously absorb two or more low‐energy photons and upconvert them into high‐energy photons through nonlinear optical processes. Yang et al.^[^ ^63^ ^]^ wrapped NaYF_4_:Yb/Er UCNPs into lead‐containing NWs generated by a solution method, and then treated them with MABr and HBr to obtain UCNP‐perovskite composite NWs (Figure 13h). Under infrared light irradiation, UCNPs can generate visible light through an energy upconversion process and are directly absorbed by the tightly surrounded perovskite NWs, thereby achieving near‐infrared detection.

## 4 Nanowire Photodetectors

### 4.1 Concept of Photodetector

The PDs discussed here are photon detectors based on internal photoelectric effects that convert optical signals into electrical signals. Photoconductive effect and photovoltaic effect are two typical types of internal photoelectric effects.

The photoconductive process refers to the phenomenon where light exposure to a semiconductor causes electrons to transition from the valence band to the conduction band, generating photogenerated charge carriers that enhance the material's conductivity. This process does not directly generate current but causes a change in conductivity. Photoconductors are the primary application of this process. Most lateral perovskite NW PDs are based on the photoconductive effect.^[^ ^32^ ^]^ Under an applied voltage, the additional photoconductivity generated by light significantly increases the current response.

**Figure 14:**
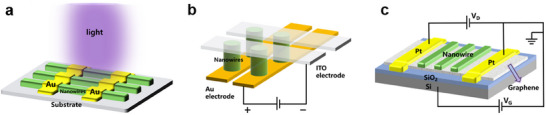
The device types of NW PDs. a) Photoconductor. b) Photodiode, referenced from Gu et al.^[^ ^186^ ^]^ c) Phototransistor, referenced from Spina et al.^[^ ^168^ ^]^

The photovoltaic process refers to the generation of electron–hole pairs in a semiconductor due to light exposure. The built‐in electric field separates these carriers, creating a potential difference across the material and generating current in the external circuit. This process can be used for photodiodes, photovoltaic devices, phototransistors, and other devices. For perovskite NWs, devices based on the photovoltaic effect can be fabricated through methods such as spin‐coating,^[^ ^65^ ^]^ using AAO membranes to grow vertical NWA,^[^ ^186^ ^]^ or by constructing perovskite HJT.^[^ ^59^ ^]^ (**Figure** **14**)

Compared to thin films or bulk single‐crystal structures, 1D NWs exhibit an extremely high surface‐to‐volume ratio, which introduces a large number of surface states. These surface states pin the Fermi level, thereby inducing band bending between the surface and the interior of the NW, which gives rise to a radial built‐in electric field and depletion region. The internal electric field facilitates the separation of photogenerated carriers, while the extended depletion region effectively suppresses the dark current.^[^ ^235^ ^]^ In addition, photogenerated carriers (particularly holes) trapped by surface states are released slowly, allowing photogenerated electrons to remain in the conduction band for an extended period.^[^ ^33^ ^]^ These effects become especially prominent under low‐light conditions: when the number of photogenerated carriers is extremely low, NW PDs can still operate effectively due to their ultralow dark current, favorable band structure for carrier separation, long carrier lifetime, and high carrier mobility. As a result, even a minute number of photogenerated carriers under weak illumination can be efficiently collected to generate a measurable photocurrent, leading to significantly enhanced detection capability in low‐light environments.

### 4.2 Performance Testing of Photodetector

#### 4.2.1 Noise

Noise sets the most fundamental limit on the detection capability of PDs. The noise in PDs primarily includes shot noise, thermal noise, and flicker noise. Shot noise is primarily induced by the dark current *i_d_*, and can be simply expressed as: $\bar{i_{sh}^{2}}=2eBi_{d},$ where *e* is the electronic charge, *B* is the system electrical response bandwidth. Thermal noise originates from the random thermal motion of electrons in a conductor. If the shunt resistance of the PD is *R_s_* ​, the mean square thermal noise power is calculated by the formula: $\bar{i_{th}^{2}}=\frac{4k_{B}TB}{R_{s}}\;,$ where *k_B_* is the Boltzmann constant and *T* is the absolute temperature. Both shot noise and thermal noise are types of white noise. Flicker noise is often called 1/f noise, and it originates from current micro electric burst pulses caused by inhomogeneities and impurities in the photosensitive layer. It mainly appears in the low‐frequency domain, below ≈1 kHz, and is inversely proportional to the modulation frequency *f* of the light radiation. If the power spectral density of 1/f noise is denoted as $\bar{i_{1/f}^{2}\left(\omega \right)}$, the total mean square noise power is given by: $\bar{i_{n}^{2}}=\bar{i_{sh}^{2}}+\bar{i_{th}^{2}}+\bar{i_{1/f}^{2}\left(\omega \right)}\cdot B$.^[^ ^236^ ^]^

#### 4.2.2 Responsivity and External Quantum Efficiency

Responsivity (*R*) is defined as the ratio of the photocurrent to the incident optical power: $R\;\left(A/W\right)=\frac{i_{ph}}{P_{in}}\;,$ where *i_ph_* is the photocurrent, *P_in_* is the incident optical power. As mentioned previously, the responsivity of perovskite NW photoconductive PDs shows significant enhancement under weak light. For this reason, when describing the responsivity performance of NWs, it is important to specify the light intensity used in the experiment.

External quantum efficiency (EQE, η_*e*_) is the ratio of the number of photogenerated charge carriers contributing to the photocurrent to the total number of incident photons, reflecting the overall efficiency of the device. The definition of EQE is given by: $\eta_{e}\;\left(\%\right)=\frac{i_{ph}/e}{P_{in}/h\upsilon }=\frac{i_{ph}hc}{P_{in}e\lambda }\;,$ where *h* is Planck constant, υ is the frequency of the incident light, *c* is the speed of light, and λ is the wavelength of the incident light. The relationship between EQE and responsivity is: $\eta_{e}=R\frac{hc}{e\lambda }\;$. For NW photoconductive PDs, since charge carriers can cycle multiple times within the device before recombining, the EQE can exceed 100%.^[^ ^34^, ^100^, ^152^ ^]^ The photoconductive gain reflects the number of photogenerated charge carriers extracted per absorbed photon. The internal photoconductive gain can also be described as $M=\frac{\tau_{0}}{\tau_{d}}\;,$ which is the ratio of the average carrier lifetime to the carrier drift time.

#### 4.2.3 Noise Equivalent Power and Detectivity

Noise equivalent power (NEP) is defined as the incident optical power required of the optical signal for the signal‐to‐noise ratio (SNR or S/N) to be unity at the detector output, that is, the *P_in_* when *i_ph_* = *i_n_* . NEP can be expressed in terms of responsivity as: $NEP\left(W\right)=\frac{i_{n}}{R}\;,$ It is worth noting that *R* here should be the responsivity when the incident optical power is as small as NEP. A smaller NEP indicates lower noise, making the PD more sensitive to weak optical signals.

Detectivity (*D*) is defined as the reciprocal of NEP: $D\;\left(W^{-1}\right)=\frac{1}{NEP}\;$. It is used to characterize the ability of a PD to generate detectable electrical signals above the noise level. A higher *D* means the PD can respond to smaller incident optical power, indicating stronger detection capability. NEP is often normalized to $\sqrt{AB}$​ to facilitate performance comparison across different detectors. The specific detectivity (D*) is obtained as: $D^{*}\;\left(\mathrm{Jones}\right)=\frac{\sqrt{AB}}{NEP}=R\frac{\sqrt{AB}}{i_{n}},$ ($1\;\mathrm{Jones}=1\;\mathrm{cm}\sqrt{\mathrm{Hz}}/W$). D* is independent of the detector's area. It is a key parameter of the intrinsic detection capability of the material and the detector structure. Since D* is defined by NEP, the responsivity used in the calculation should correspond to the value at the incident optical power corresponding to NEP. If *R* is taken at another power level, the linear dynamic range (LDR) should cover the power range from NEP to the testing point. In fact, for perovskite NW PDs, since the responsivity increases significantly at low light conditions, the calculated NEP and D* may deviate from their true significance. Nevertheless, D* remains a crucial parameter for performance comparison across detectors.

#### 4.2.4 Linear Dynamic Range

Linearity reflects the linear relationship between the photocurrent and the incident optical power, with the slope being the responsivity. PDs require linear response to ensure that the output electrical signal remains undistorted relative to the input optical signal. When a PD exhibits a linear response, the η_*e*_​ and *R* are constants independent of *P_in_* ​, with the LDR showing a slope of unity on a log‐log plot of *i_ph_* versus *P_in_*. As the *P_in_* increases beyond the saturation signal power (*P_sat_*), the PD's optical response starts to saturate, reaching *I_sat_* ​. LDR is usually quantified in decibels (dB) as: $LDR\;\left(dB\right)=10\;\log\frac{P_{sat}}{NEP}=10\log\frac{I_{sat}}{i_{n}}\;$. In practical measurements, if the photocurrent is found to increase linearly within the dynamic optical power range from *P_min_* to *P_max_*, the measured LDR is: $LDR\;\left(dB\right)=10\;\log\frac{P_{max}}{P_{min}}=10\log\frac{I_{max}}{I_{min}}$. It is important to note that for perovskite NW PDs, since *R* increases significantly at low light intensities, the slope in the log‐log plot of the LDR will be less than 1. In this case, the linear relationship between the logarithmic values has deviated from the original meaning of linearity and is shown merely as a reference indicator. Most literature uses the formula: $LDR\;\left(dB\right)=20\;\log\frac{P_{max}}{P_{min}}=20\log\frac{I_{max}}{I_{min}}\;$ as a measure for NW detectors. A careful comparison between the LDRs of different devices needs to be carried out to avoid confusion between different logarithmic factors.^[^ ^237^ ^]^

#### 4.2.5 Response Speed

The response speed of a PD determines the ability of a PD to follow a fast‐varying optical signal. It can be described from both the time domain (response time) and frequency domain (−3 dB bandwidth). The response time of a PD must be shorter than the fastest time variation in the signal, or equivalently, the PD should have a frequency response that covers the entire bandwidth of the signal. In the time domain, the response speed is typically characterized by the rise time *t_r_* ​ and fall time *t_f_* ​, which describe the response of detector to a pulse or square wave signal. The rise time is defined as the time interval for the response to increase from 10% to 90% of its peak value, while the fall time is defined as the time interval for the response to decay from 90% to 10% of its peak value. In the frequency domain, the response speed of a PD is usually characterized by its −3 dB bandwidth, which is the modulation frequency at which the responsivity of the device is half of that under steady‐state conditions. This quantity depends on the carrier transit time (*t_tr_*) and the resistor–capacitor (RC) time constant of the circuit: $f_{-3dB}^{2}={\left(\frac{3.5}{2\pi t_{tr}}\right)}^{2}+{\left(\frac{1}{2\pi RC}\right)}^{2}\;$.^[^ ^237^ ^]^

#### 4.2.6 Carrier Mobility and Trap Density

**Figure 15:**
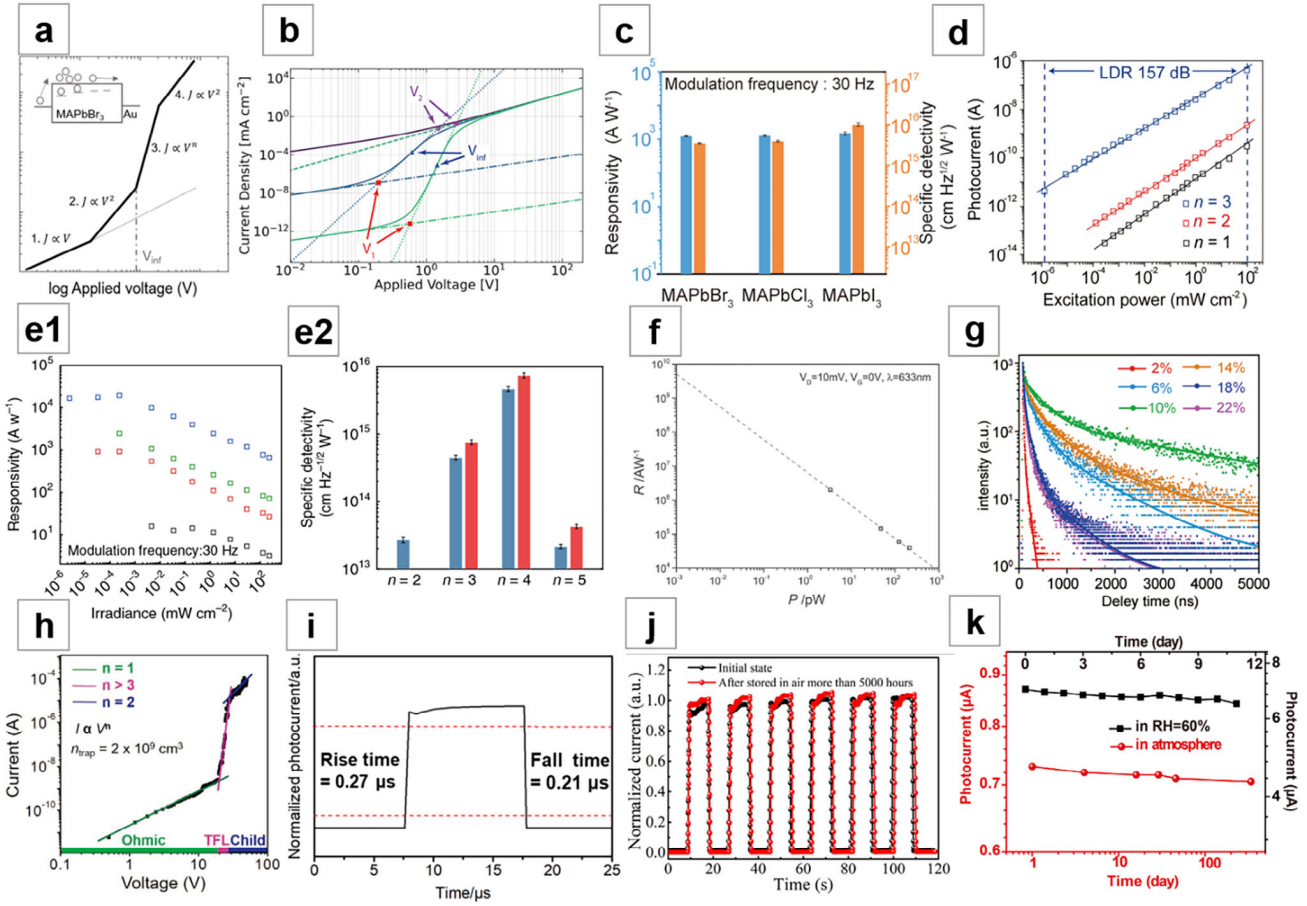
Performance of NW PDs. a) SCLC behavior for a semiconductor with trap states. Reproduced with permission.^[^ ^238^ ^]^ Copyright 2020, American Chemical Society. b) Simulated J–V curve. Reproduced under terms of the CC‐BY‐NC‐ND license.^[^ ^239^ ^]^ Copyright 2021, Vincent M. Le Corre et al., published by American Chemical Society. c) Ultrahigh D* of MAPbX_3_ (X = Br, Cl, I) MWA. Reproduced with permission.^[^ ^39^ ^]^ Copyright 2023, John Wiley and Sons. d) Large LDR. Reproduced with permission.^[^ ^38^ ^]^ Copyright 2020, John Wiley and Sons. e) e1: R and e2: D* of the advanced 2D perovskite NWA PD. Reproduced with permission.^[^ ^32^ ^]^ Copyright 2018, Jiangang Feng et al., published by Springer Nature. f) Ultrahigh responsivity of MAPbI_3_ NW/graphene devices. Reproduced with permission.^[^ ^37^ ^]^ Copyright 2015, John Wiley and Sons. g) Long carrier lifetime. Reproduced with permission.^[^ ^178^ ^]^ Copyright 2020, John Wiley and Sons. h) Ultralow trap density. Reproduced with permission.^[^ ^170^ ^]^ Copyright 2020, John Wiley and Sons. i) Ultrafast response times. Reproduced with permission.^[^ ^69^ ^]^ Copyright 2021, John Wiley and Sons. j) Normalized I–t curves before and after stored in the air environment for more than 5000 h. Reproduced under terms of the CC‐BY license.^[^ ^73^ ^]^ Copyright 2022, Dingjun Wu et al., published by John Wiley and Sons. k) Variation in the photocurrent of the PD with exposure time in air and at RH = 60%. Reproduced with permission.^[^ ^173^ ^]^ Copyright 2020, John Wiley and Sons.

For semiconductors with trap states, the space‐charge‐limited current (SCLC) method is commonly used to measure carrier mobility and trap density (**Figure** **15**a).^[^ ^238^ ^]^ When excess carriers are injected into a medium with relatively low carrier mobility, some of the carriers remain in the medium, forming space charges that impede further charge injection. The current density in this regime follows the Mott‐Gurney law: $J_{SCLC}=\frac{9}{8}\;\varepsilon \mu \frac{V^{2}}{L^{3}},$ where ε is the dielectric constant of the material, µ is the mobility, *L* is the thickness of the medium, *V* is the applied voltage, and *J* is the current density. Carrier mobility can be calculated from the current density in the SCLC region. The trap density (*n_t_*) is commonly measured using the trap‐filled‐limit voltage (*V_TFL_*): $n_{t}=\frac{2\varepsilon V_{TFL}}{eL^{2}}\;$. However, there is no clear consensus on which point of the J–V curve corresponds to *V_TFL_*. According to the report by Corre et al.,^[^ ^239^ ^]^ using the tangent intersection voltage *V*_2_ of slope 2 and slope >2 is the most accurate method (Figure 15b).

### 4.3 Performance of Nanowire Photodetectors

Since the first perovskite NW PD was fabricated by Horváth et al.,^[^ ^40^ ^]^ high‐performance, fast‐response, and ultra‐stable perovskite NW PDs have been reported. The following **Table** **2** (provided at the end of the article) shows the R, D* and corresponding test conditions, response speed, stability and other information of some detectors arranged by D*.

**Table 2:** Performance of NW PDs.

Structure and synthesis method	R [A W^−1^]	D*[Jones]	Response speed	Long‐term stability	Refs.
MAPbI_3_ MWA (Wettability‐guided)	1490 @2.5 nW cm −^2^, 5 V	9.77 × 10^15^	278/356 µs	almost stable @10 days	[39]
(ThMA)_2_(MA)_n‐1_Pb_n_I_3n+1_ (n = 3) NWA (Wettability‐guided)	1100 @1.27 nW cm‐^2^,5 V	9.1 × 10^15^	36.2/31.5 µs	–	[38]
(BA)_2_(MA)_n‐1_Pb_n_I_3n+1_ (n = 4) NWA (Wettability‐guided)	1.5 × 10^4^ @2.33 nW cm‐^2^, 5 V	7 × 10^15^	27.6/24.5 µs	–	[32]
MAPbI_3_ NW networks/graphene (Slip‐coating)	2.6 × 10^6^ @0.65 nW cm‐^2^, 10 mV	≈ 1 × 10^15^	55/75 s	–	[37]
(BA)_2_(MA)_n‐1_Pb_n_Br_3n+1_ (n = 4) MWA (Si‐template‐guided)	3.5 @0.079 mW, 5 V	1 × 10^15^	4.1/3.3 ms	slight decline @5 days, 60%RH	[172]
CsPbI_3_ NWA with PVP (Wettability‐guided)	1294 @0.53 µW cm‐^2^, 5 V	2.6 × 10^14^	0.85/0.78 ms	90% remain @30 days	[177]
CsPbI_3_ NW (VLS)	1350 @5 mW cm‐^2^, 1 V	1.8 × 10^14^	120 µs	–	[134]
FAPbI_3_ NWA with PEA^+^ (Wettability‐guided)	5282 @44 nW cm‐^2^, 5 V	1.45 × 10^14^	29.3/31.1 µs	90% remain @28 days, 50%RH	[178]
MAPbI_3_ NW networks (Dip‐coating)	8500 @5.5 nW cm‐^2^, 1 V	1.2 × 10^14^	350/670 µs	70% remain @20 days, 10%RH	[34]
MAPbBr_3_ NWA (Film Confinement)	1026.5 @154 nW cm‐^2^, 1 V	1.05 × 10^14^	3.0/2.3 ms	95% remain @223 days	[152]
MAPbI_3_ NW networks with DPP‐CNTVT (Spin‐coating)	0.5 @0 V	9.51 × 10^13^	0.27/0.21 µs	90% remain @15 300 h, 65%RH	[69]
MAPbBr_3_ NWA (Film Confinement)	15.62 @3.25 mW cm‐^2^, 5 V	5.58 × 10^13^	1.12/0.63 ms	–	[151]
CsPbI_3_ nanorod (Colloidal Method)	2920 @10.69 mW cm‐^2^, 2 V	5.17 × 10^13^	0.05/0.15 ms	almost stable @one week	[100]
MAPbBr_3_ NWA (Wettability‐guided)	686 @0.13 µW cm‐^2^, 5 V	4.73 × 10^13^	41.2/48.9 µs	–	[183]
CsPbI_3_ NWA (Blade‐coating)	1470	4.1 × 10^13^	–	–	[77]
MAPbI_3_/photonic crystal NWA (Film Confinement)	12.67 @32 mW cm‐^2^, 10 V	3.22 × 10^13^	21/67 ms	76.2% remain @30 days	[155]
MAPbI_3_ NWA (Film Confinement)	125.2 @5 µW cm‐^2^, 5 V	2.8 × 10^13^	–	–	[147]
MAPbI_3_‐MAPbBr_3_ MWA HJT (Template‐guided)	1207 @1.18 nW cm‐^2^, 5 V	2.78 × 10^13^	3.9/2.0 ms	88.2% remain @391 days	[174]
MAPbI_3_ NW (Antisolvent‐assisted)	460 @60 µW cm‐^2^, 5 V	2.6 × 10^13^	0.18/0.33 ms	slight decline @45 days, 30∼45%RH	[81]
MAPbI_3_ NW networks with BMIMBF_4_ (Spin‐coating)	37.14 @1.45 nW cm‐^2^, 5 V	2.06 × 10^13^	91/563 µs	almost stable @5000 h, 35%RH	[73]
MAPbI_3_ NW networks with OA (Drop‐coating)	4.95 @1 V	2 × 10^13^	0.1/0.1 ms	94% remain @30 days	[44]
MAPbI_3_ NWA (Template‐guided)	118 @ ≈ 2 nW cm‐^2^, 5 V	1.95 × 10^13^	152/206 µs	–	[175]
CsPbCl_3_/CsPbI_3_ NW superlattices (VLS)	49 @2.11 mW cm‐^2^, 5 V	1.51 × 10^13^	13/16 ms	–	[231]
(R‐/S‐α‐PEA)_2_PbI_4_ NWA (Wettability‐guided)	47.1 @150 nW cm‐^2^, 5 V	1.24 × 10^13^	267/258 µs	XRD stable @30 days, 45%RH	[36]
MAPbI_3_ NW (Microfluidic)	410 @5 mW cm‐^2^, − 1 V	9.1 × 10^12^	0.22/0.79 ms	XRD stable @100 days, 40%RH	[166]
CsPbI_3_ NW (VLS)	4489 @0.2 nW cm‐^2^	7.9 × 10^12^	–	–	[132]
FAPbI_3_ NWA (DLW)	11.7 @60 µW cm‐^2^, 5 V	7.8 × 10^12^	–	–	[140]
MAPbI_3_ NW networks with Pb(SCN)_2_ (Spin‐coating)	0.62 @100 mW cm‐^2^, 10 V	7.3 × 10^12^	227.2/215.4 µs	98% remain @30 days, 40%RH	[74]
MAPbI_3_ NWA with P(VDF‐TrFE) (Film Confinement)	0.0125 @25.4 µW cm‐^2^, 0 V	7.3 × 10^12^	88/154 µs	–	[153]
MAPbI_3_ NWA (Blade‐coating)	6660 @0.12 µW cm‐^2^, 2 V	6.85 × 10^12^ @3 µW cm‐^2^	–	–	[170]
CsPbBr_3_ NW networks (Colloidal Method)	0.00236 @0.6 mW cm‐^2^, 5 V	6.17 × 10^12^	3/2.8 ms	–	[101]
MAPbI_3_ MWA (Blade‐coating)	13.57 @500 µW cm^−2^, − 5 V	5.25 × 10^12^	80/240 µs	slight decline @50 days, 45∼55%RH	[35]
MAPbI_3_ NWA (Film Confinement)	20.56 @2.2 µW cm‐^2^, 1 V	4.73 × 10^12^	–	–	[157]
MAPbI_3_ NW networks (Spin‐coating)	0.56 @145 mW cm‐^2^, 5 V	4.16 × 10^12^	0.2/0.37 ms	80% remain @30 days, 55∼65%RH	[67]
CsPbBr_3_ MWA (Vapor‐phase synthesis)	7.66 @1.02 mW cm‐^2^, − 5 V	4.05 × 10^12^	275/550 ms	–	[207]
MAPbI_3_ NWA (Dip‐coating)	13.85 @5 µW cm‐^2^, 10 V	3.87 × 10^12^	50/50 ms	90% remain @90 days, 45∼55%RH	[64]
MAPbI_3_ NW networks (Drop‐coating)	1.3 @80 µW cm^−2^, 30 V	2.5 × 10^12^	0.2/0.3 ms	–	[41]
MAPbI_3_ NW networks (Inkjet Printing)	1.2 @0.1 mW cm‐^2^, 10 V	2.39 × 10^12^	10/10 ms	–	[143]
MAPbI_3_ NW networks with C8BTBT (Spin‐coating)	8.1 @7.5 µW cm‐^2^, 3 V	2.17 × 10^12^	7.1/6.5 ms	70% remain @50 days, 45%RH	[70]
CsCu_2_I_3_ NW (Antisolvent‐assisted)	32.3	1.89 × 10^12^	6.94/214 µs	–	[87]
MAPbI_3_ NW networks (Drop‐coating)	0.1611 @9 µW cm‐^2^, 0 V	1.3 × 10^12^	13.8/16.1 µs	–	[46]
MAPbI_3_ MWA (Blade‐coating)	0.48 @22.9 mW cm‐^2^, 2 V	1.26 × 10^12^	36/43 ms	83% remain @2 days, 50∼60%RH	[79]
MAPbI_3_ NW networks (Drop‐coating)	0.1 @10 V	1.02 × 10^12^	0.3/0.4 ms	XRD stable @30 days, 70%RH	[42]
MAPbI_3_ NW networks with Pb(SCN)_2_ (Spin‐coating)	0.23 @100 mW cm‐^2^, 2 V	7.1 × 10^11^	53.2/50.2 µs	98% remain @500 h, 50%RH	[71]
MAPbI_3_ NW (Blade‐coating)	0.04	6 × 10^11^	178/173 µs	slight decline @60 days, 45∼55%RH	[78]
MAPbBr_3_ MWA (Template‐guided)	20 @6.37 nW cm^−2^, 1 V	4.1 × 10^11^	1.6/6.4 ms	96% remain @480 days	[173]
Cs_0.5_MA_0.5_PbI_3_ NW (Solution synthesis)	23 @1.5 mW cm‐^2^, 5 V	2.5 × 10^11^	–	–	[43]
MAPbI_3_ NWA (Spin‐coating)	0.0022 @0 V	1.76 × 10^11^	27.2/26.2 ms	–	[169]
MAPb(I_1‐x_Br_x_)_3_ NWA (Antisolvent‐assisted)	12 500 @42 µW cm‐^2^, 5 V	1.73 × 10^11^	0.34/0.42 µs	slight decline @13 days, 45∼55%RH	[184]
CsPb(Br_1−x_I_x_)_3_ NW (Vapor‐phase synthesis)	0.32936 @0.45 mW cm‐^2^, 2 V	≈ 1 × 10^11^	25/29 ms	–	[85]
MASnI_3_ NWA (AAO‐guided)	0.47 @1.1 mW cm‐^2^, 5 V	8.8 × 10^10^	1500/400 ms	14% remain @7 days, 70%RH	[190]
CsPbI_3_ NW networks (Drop‐coating)	0.745 @0.282 mW cm‐^2^	3.46 × 10^10^	–	–	[45]
MAPbI_3_ NWA (AAO‐guided)	0.03 @0.3 V	1 × 10^10^	20.47/13.81 ms	–	[186]
FAPbI_3_ NWA (AAO‐guided)	0.3032 @0.3 µW cm‐^2^	1.1 × 10^9^	19.2/23.9 ms	–	[22]
CsPbI_3_ NWA (AAO‐guided)	0.0067 @1.5 mW cm‐^2^	1.57 × 10^8^	0.292/0.234 s	–	[189]
CsPbBr_3_ NWA (Sapphire Epitaxy)	4400 @0.2 mW cm‐^2^, 3 V	–	252/300 µs	–	[123]
MAPbI_3_ NWA (Wettability‐guided)	3160 @0.0177 nW, 5 V	–	–	–	[179]
MAPbBr_3_ NWA (Wettability‐guided)	1377 @0.0346 nW	–	21.5/23.4 µs	–	[176]
MAPbBr_3_‐ MAPbBr_3‐x_I_x_ NWA HJT (Wettability‐guided)	265 @14 µW cm‐^2^, 5 V	–	170.5/91.3 ms	90% remain @10 days	[182]
CsPbBr_3_ NWA‐IGZO HJT (Mica Epitaxy)	3.794 @2.93 mW cm‐^2^, − 5 V	–	2/94 ms	slight decline @60 days	[121]
CsPbBr_3_ NW (AAO‐guided)	1.9 @40 mW cm‐^2^, 5 V	–	0.2 s	–	[161]
MAPbI_3_ NWA (Microfluidic)	0.2 @1 mW cm‐^2^, 5 V	–	50/50 ms	–	[167]
CsPbI_3_‐CsPbBr_3_ NWA HJT (Film Confinement)	0.125 @0.5 µW cm‐^2^, 0 V	–	0.7/0.8 ms	85% remain @15 days	[150]
MAPbI_3_ NW networks (Slip‐coating)	0.005 @70 nW cm‐^2^, 1 V	–	0.35/0.25 ms	–	[40]
(R/S‐NEA)PbI_3_ NWA (Film Confinement)	≈0.003	–	32.62/26.73 µs	–	[156]
MAPbI_3_ QWA (AAO‐guided)	0.002 @ − 5 V	–	80/140 ms	–	[191]

The perovskite NWA grown by asymmetric‐wettability template‐assisted method exhibit high crystalline quality. Numerous detectors fabricated from these NWs demonstrate ultrahigh detectivity exceeding 10^15^ Jones. The D* of MAPbBr_3_, MAPbCl_3_, and MAPbI_3_ MWA synthesized by Fu et al.^[^ ^39^ ^]^ reached 3.42 × 10^15^, 3.84 × 10^15^, and 9.77 × 10^15^ Jones, respectively, representing the highest values reported to date. The responsivities for all three materials also exceeded 10^3^ A W^−1^ (Figure 15c). Zhao et al.^[^ ^38^ ^]^ synthesized the (ThMA)_2_(MA)_n‐1_PbI_3n+1_ NWA (ThMA is 2‐thiophenemethylamine), which achieved a D* of 9.1 × 10^15^ Jones, with response times of 36.2/31.5 µs and a LDR of 157 dB (Figure 15d). Feng et al.^[^ ^32^ ^]^ also synthesized the (BA)_2_(MA)_n‐1_Pb_n_I_3n+1_ NWA, achieving a responsivity of 1.5 × 10^4^ A W^−1^, D* of 7 × 10^15^ Jones, and a fast response time of 27.6/24.5 µs, which was the most advanced PDs at the time (Figure 15e). Mechanistically, this can be attributed to the full utilization of the superior properties of NWs: their small linewidth and the presence of edge states in 2D perovskite provide a direct pathway for exciton dissociation. The resulting free carriers are trapped in these edge states, which prolongs their lifetimes. Meanwhile, the serial hopping barriers formed by the organic layers within the crystal structure of 2D perovskite NWs further suppress the dark current. This combination of high internal resistance and high edge conductivity leads to reduced dark current and enhanced photocurrent, enabling exceptional detection performance under weak illumination. An increasing number of high‐detectivity devices continue to demonstrate that the lower the incident light power, the more outstanding the performance of NW PDs becomes.^[^ ^32^, ^34^, ^37^, ^38^, ^39^, ^134^, ^152^, ^172^, ^177^, ^178^ ^]^ Spina et al.^[^ ^37^ ^]^ deposited MAPbI_3_ NW networks onto a single‐layer graphene FET using the spin‐coating method, creating a NW/graphene phototransistor. Due to the excellent electronic characteristics of graphene and the photo‐doping/gating effect, the responsivity was significantly enhanced, reaching 2.6 × 10^6^ A W^−1^ at an optical power of 3.3 pW (0.65 nW mm^−2^). The sharp increase in responsivity at low light intensities demonstrates the potential of perovskite NW/graphene devices for low‐light imaging sensors and single‐photon detectors (Figure 15f).

Chen et al.^[^ ^178^ ^]^ reported that the FAPbI_3_ NWA grown by asymmetric‐wettability template exhibited a PL lifetime of 2023.3 ns, indicating suppressed trap density and high crystallinity (Figure 15g). Wu et al.^[^ ^67^ ^]^ fabricated a welded NW networks via spin‐coating, which also exhibited a carrier lifetime of up to 1363 ns. Deng et al.^[^ ^170^ ^]^ obtained MAPbI_3_ NWA by blade‐coating onto templates, achieving an ultralow trap density of 2 × 10^9^ cm^−3^ (Figure 15h). Dai et al.^[^ ^47^ ^]^ fabricated NW networks by drop‐coating, which exhibited a low trap density of 5 × 10^10^ cm^−3^ and a carrier mobility exceeding 600 cm^2^ (V·s)^−1^, but applied to photovoltaic devices. The device reported by Chang et al.^[^ ^69^ ^]^ exhibits the fastest response speed (0.27/0.21 µs) (Figure 15i), the highest LDR (265 dB), and the longest long‐term stability (15300 h, 638 days), which was achieved by spin‐coating DPP‐CNTVT as a multifunctional interfacial layer onto the MAPbI_3_ NW network. Deng et al.^[^ ^184^ ^]^ synthesized MAPb(I_1‐x_Br_x_)_3_ NWA via antisolvent vapor‐assisted synthesis, which exhibited ultrafast response times of 0.34/0.42 µs and a long carrier diffusion length of 41 µm. Oksenberg et al.^[^ ^126^ ^]^ also fabricated devices with response times of 5.2/3.2 µs, but did not mention further tests on the detection performance. Chen et al. incorporated an appropriate amount of PEA^+^ cations^[^ ^178^ ^]^ or PVP^[^ ^177^ ^]^ into the perovskite solution, enabling the fabricated NWA to retain 90% of their initial performance after one month. Gao et al.^[^ ^44^ ^]^ fabricated NW devices passivated with OA via spin‐coating, which maintained 94% of their performance after being stored in air for one month. Wu et al.^[^ ^73^ ^]^ introduced the ionic liquid BMIMBF_4_ into MAPbI_3_ NW, passivating defects and suppressing perovskite degradation. After 5000 h (208 days) of exposure to air, the performance showed no degradation, indicating exceptional long‐term stability (Figure 15j). Encapsulation can further enhance the stability of NW. Li et al. demonstrated that devices wrapped in PDMS retained 95.7% of their performance after continuous exposure to water vapor for 3 h.^[^ ^152^ ^]^ Devices in situ encapsulated in FOTS maintained 96% of their initial performance even after 480 days (Figure 15k).^[^ ^173^ ^]^ It is worth noting that unencapsulated devices still maintained 88.2% of their photocurrent after being exposed to air for 391 days, which was attributed to the improved crystal quality achieved through the fabrication process.^[^ ^174^ ^]^

Nontoxic, lead‐free perovskites have consistently been a major focus in the push toward commercialization. However, the most commonly used alternative element, tin, is easily oxidized. Moreover, lead‐free perovskites often suffer from challenges such as difficult nucleation, high defect density, severe non‐radiative recombination, and unfavorable band structures and charge transport properties, all of which limit their optoelectronic performance. Consequently, reports on lead‐free perovskite NWs remain relatively scarce. Waleed et al.^[^ ^190^ ^]^ synthesized MASnI_3_ perovskites using a porous alumina template, which offered excellent encapsulation, effectively suppressed the lateral diffusion of water and oxygen molecules, and helped prevent lattice distortion. The resulting PD exhibited a detectivity of 8.8 × 10^10^ Jones and maintained stability for 7 days under high humidity. It is expected that the development of more advanced encapsulation and passivation strategies will continue to enhance the performance of lead‐free perovskite NW devices.

It is worth mentioning that perovskite NWs have also been employed for X‐ray detection. Kundu et al.^[^ ^240^ ^]^ demonstrated CsPbI_3_ NW with a sensitivity of 190 µCGy_air_^−1^cm^−2^ and a detection limit of 33.3 nGy_air_/s. Further reports on both direct and indirect X‐ray NW detectors have also emerged.^[^ ^241^, ^242^, ^243^, ^244^ ^]^ However, due to the limitations in the morphology of NWs, it remains challenging to fabricate high‐performance radiation detectors.

## 5 Application of Nanowire Photodetectors

### 5.1 Polarization Application

Polarized light refers to light whose electric field vector exhibits a specific direction or regular pattern along the propagation direction, and it can be classified as linear polarized light (LPL), circularly polarized light (CPL), and elliptically polarized light. NWs can generate strongly polarized PL emission and can also be used to detect the dichroism of light with different polarization states. When NWs generates polarized light, the degree of polarization (DOP) is used to characterize the extent of polarization, given by: $P=\frac{I_{max}-I_{min}}{I_{max}+I_{min}}\;,$ where *I_max_* ​ and *I_min_* ​ are the light intensities at the strongest and weakest polarization directions, respectively. When NWs detects LPL, the anisotropy ratio (dichroism ratio) $\omega =\frac{I_{M}}{I_{m}}\;$​​ reflects the difference in photocurrent response of the detector to different polarization directions of LPL, where *I_M_* and *I_m_* ​ are the maximum and minimum photocurrents for different linear polarization directions, respectively. For chiral NWs detecting CPL, the anisotropy factor $g_{I_{ph}}=2\frac{I_{R}-I_{L}}{I_{R}+I_{L}}\;$ is usually used to reflect the difference in photocurrent response of the detector to LCP and RCP, where *I_L_* and *I_R_* ​ are the photocurrents generated by LCP and RCP, respectively.

**Figure 16:**
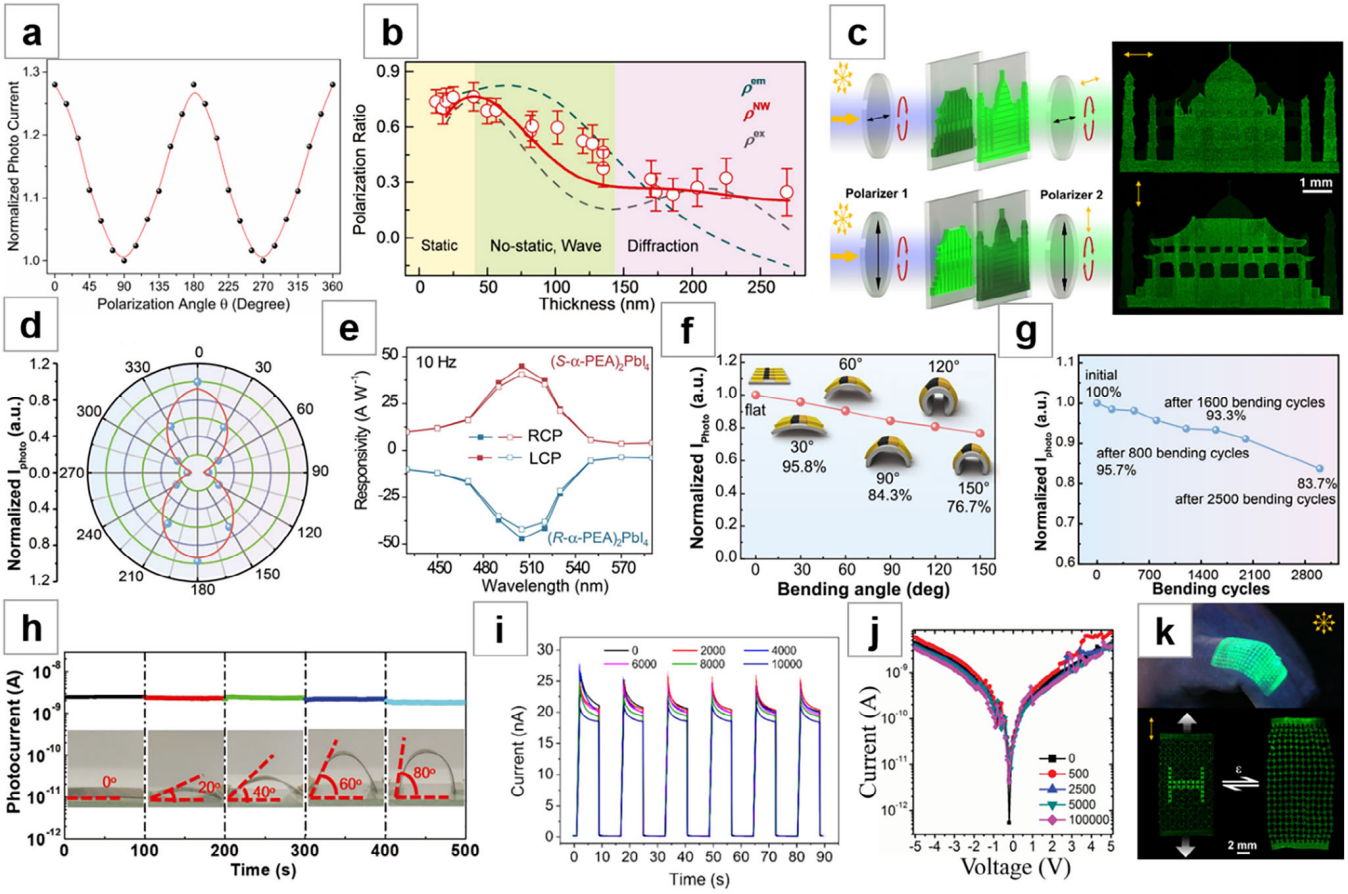
Polarization application and flexible devices. a) Perovskite NW PDs response to LPL. Reproduced with permission.^[^ ^44^ ^]^ Copyright 2016, American Chemical Society. b) The emission polarization ratio of the NWs undergoes three regimes with the increase of NW thickness. Reproduced with permission.^[^ ^117^ ^]^ Copyright 2018, John Wiley and Sons. c) Viewing different patterns through polarizers. Reproduced under terms of the CC‐BY‐NC license.^[^ ^98^ ^]^ Copyright 2019, Nanjia Zhou et al., published by The American Association for the Advancement of Science. d) High polarization ratio polarization‐sensitive PD. Reproduced with permission.^[^ ^152^ ^]^ Copyright 2022, John Wiley and Sons. e) Different responsivities of the (R‐α‐PEA)_2_PbI_4_ NW device under RCP and LCP illumination. Reproduced with permission.^[^ ^36^ ^]^ Copyright 2021, American Chemical Society. f) Photocurrent for different bending states. Reproduced with permission.^[^ ^174^ ^]^ Copyright 2022, John Wiley and Sons. g) Photocurrent after different bending cycles. Reproduced with permission.^[^ ^152^ ^]^ Copyright 2022, John Wiley and Sons. h) Photographs of the devices under the different bending degrees. Reproduced with permission.^[^ ^64^ ^]^ Copyright 2018, John Wiley and Sons. i) I–t curve evolution at different bending cycles. Reproduced with permission.^[^ ^42^ ^]^ Copyright 2015, American Chemical Society. j) I–V curves of the super flexible PD. Reproduced with permission.^[^ ^35^ ^]^ Copyright 2016, John Wiley and Sons. k) Flexible structure adheres to a finger. Reproduced under terms of the CC‐BY‐NC license.^[^ ^98^ ^]^ Copyright 2019, Nanjia Zhou et al., published by The American Association for the Advancement of Science.

Täuber et al.^[^ ^52^ ^]^ investigated the polarization properties of PL emission and excitation in single MAPbI_3_ NW of different temperatures and physical shapes. They achieved a maximum PL emission polarization ratio of 0.7, while PL excitation was nearly unpolarized. No correlation was found between the DOP and the aspect ratio of the NWs. The high PL emission polarization was attributed to the asymmetric and distorted crystal structure of the unit cell. Gao et al.^[^ ^44^ ^]^ demonstrated, for the first time, the response of perovskite NW detectors to LPL. The devices exhibited peaks and valleys in photocurrent at angles where the light was polarized parallel and perpendicular to the NW orientation axis, with an anisotropy ratio of 1.3 (**Figure** **16**a). Zhou et al.^[^ ^45^ ^]^ from the same group grew orthorhombic β‐CsPbI_3_ NW, which exhibited high sensitivity to LPL due to anisotropies in both morphology and crystal structure. The photocurrent anisotropy ratios on rigid and flexible substrates were 2.68 and 2.17, respectively. Ghoshal et al.^[^ ^110^ ^]^ synthesized (BA)_2_PbI_4_ 2D NW via vapor deposition, achieving a PL emission polarization ratio of 0.73. The NW/graphene hybrid device showed a photocurrent anisotropy ratio of 3.62. Gao et al.^[^ ^117^ ^]^ grew CsPbBr_3_ and CsPbCl_3_ ultrathin NWs on muscovite mica substrates by the Waals epitaxial method. As the NW thickness increased, the emission polarization ratio underwent three regimes of nearly invariant, decreasing, and finally oscillating in small amplitude, which were owing to electrostatic mismatch, enhancement of light wave characteristics, and competition between multi‐waveguide modes, respectively. The highest DOP reached ≈0.78 (Figure 16b). Lin et al.^[^ ^163^ ^]^ fabricated perovskite NWA using an AAO template, and produced polarized emission with a DOP of up to 0.84 using blue LED light. Zhou et al.^[^ ^98^ ^]^ dispersed CsPbBr_3_ NW bundles, grown through a colloidal method, in the toluene solution of polystyrene–polyisoprene–polystyrene (SIS) as ink. Using a unique direct ink writing technique developed by their team,^[^ ^245^ ^]^ they demonstrated the charm of polarized emission, including obtaining different grayscale intensities by orienting the nanocomposites in various directions, viewing different patterns through polarizers, and performing display/encryption via reversible stretching motions (Figure 16c).

Feng et al.^[^ ^176^ ^]^ fabricated CsPbBr_3_ NWA using the asymmetric‐wettability template, achieving an anisotropy ratio of 2.6. Li et al.^[^ ^87^ ^]^ synthesized CsCu_2_I_3_ copper halide perovskite NW, which demonstrated excellent polarization detection capabilities in the ultraviolet range of 230 to 350 nm, with an anisotropy ratio of 3.16. Li et al.^[^ ^174^ ^]^ used a template‐guided method to synthesize MAPbBr_3_‐MAPbI_3_ MWA HJT, which exhibited a high anisotropy ratio of up to 8.2. Subsequently,^[^ ^152^ ^]^ using two‐layer PDMS templates with a relative rotation angle θ, they synthesized MAPbBr_3_ NWA with a Moiré lattice structure, achieving an anisotropy ratio as high as 9.1(Figure 16d). Zhao et al.^[^ ^36^ ^]^ synthesized (S‐α‐PEA)_2_PbI_4_ and (R‐α‐PEA)_2_PbI_4_ perovskite NWA with different chiral molecules added through an asymmetric‐wettability template, which not only showed an anisotropy ratio of 1.6 for LPL, but also exhibited significant differences under LCP and RCP illumination, with an anisotropy factor for responsivity of 0.15. Since chiral perovskite NWs respond to both LPL and CPL, full Stokes parameter PDs were fabricated (Figure 16e). Liu et al.^[^ ^181^ ^]^ synthesized (R‐/S‐MBA)_2_PbI_4_ NWA using a similar method, which was also suitable for detecting CPL, with $g_{I_{ph}}$ of 0.24. Wang et al.^[^ ^156^ ^]^ imprinted (R/S‐NEA)PbI_3_ chiral perovskite thin films into NWA and found that the grating structure in the form of NWs was effective for detecting LPL, while the film has a more prominent effect on CPL. Therefore, a partial imprinting strategy was adopted to achieve a balanced recognition effect on CPL and LPL and construct a Stokes detector. In addition, many other NWs synthesized through various methods also demonstrated excellent polarization emission and detection capabilities.^[^ ^94^, ^151^, ^157^, ^169^, ^172^, ^208^, ^228^ ^]^

### 5.2 Flexible Devices

The 1D NW structure of perovskites provided superior mechanical flexibility, making NW detectors ideal for integration into flexible components of wearable devices. Li et al.^[^ ^174^ ^]^ demonstrated that their template‐guided MAPbI_3_‐MAPbBr_3_ MWA HJT retained 83.3% of their original performance after 3000 bending cycles. Even with a bending angle of 150°, the device's photocurrent remains 76.7% of that in the flat state (Figure 16f). Li et al.^[^ ^152^ ^]^ also used two layers of PDMS to in situ encapsulate a moiré lattice NWA PD. Due to the excellent mechanical properties of PDMS, the detector exhibited remarkable flexibility. When the device was bent to a 150° angle, its light response maintained 82.4% of its original value, and after 2000 bending cycles at a 60° angle, the photocurrent was still 91.1% of the initial value (Figure 16g). Hu et al.^[^ ^175^ ^]^ fabricated MAPbI_3_ NWA using a template‐guided method and tested it under a 6 mm bending radius for 2000 cycles, maintaining 87% of its performance. Chen et al.^[^ ^64^ ^]^ fabricated NW devices using a dip‐coating method, observing only a 5% decrease in photocurrent after 5000 bending cycles (Figure 16h). Similarly, NW devices fabricated by drop‐coating^[^ ^42^ ^]^ maintained 90% of their photocurrent after 10000 cycles at a fixed 40° bending angle (Figure 16i). Deng et al.^[^ ^35^ ^]^ used a blade‐coating method to fabricate MWA, and even after 10^5^ bending cycles, the light response showed only a slight decrease (Figure 16j). Zhou et al.^[^ ^98^ ^]^ demonstrated an interesting example by attaching a CsPbBr_3_ NW‐SIS flexible structure adhere to a finger, where the polarization emission patterns from the NWs could be either displayed or hidden through reversible stretching motions (Figure 16k). More flexible devices have been demonstrated.^[^ ^39^, ^45^, ^67^, ^70^, ^74^, ^79^, ^87^, ^147^, ^150^, ^153^, ^166^, ^173^ ^]^

### 5.3 Image Sensors

**Figure 17:**
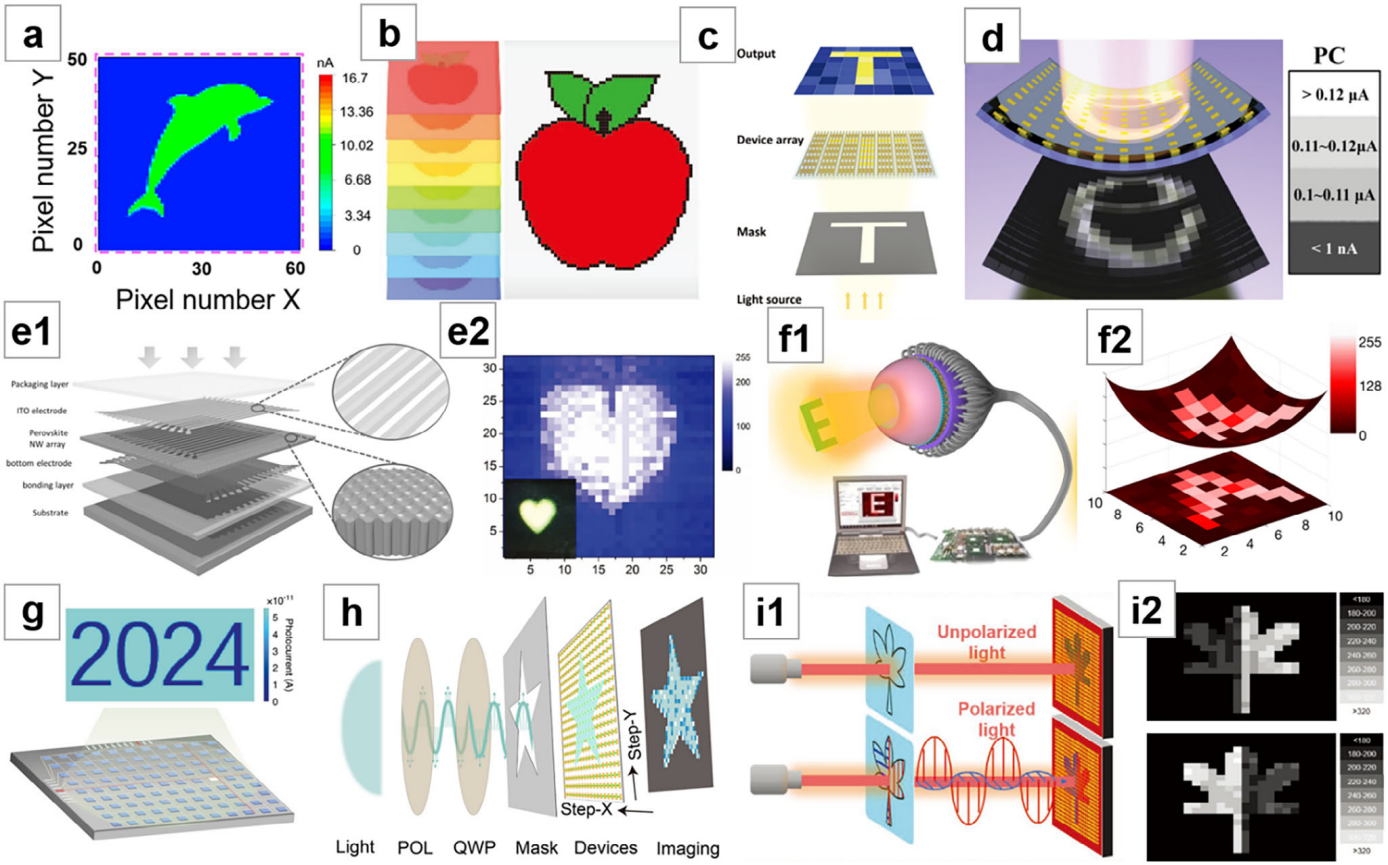
NW for image sensors. a) 2D photocurrent mapping of the image of “Dolphin”. Reproduced with permission.^[^ ^231^ ^]^ Copyright 2024, American Chemical Society. b) Pseudo‐colored image converted from the spectra. Reproduced with permission.^[^ ^229^ ^]^ Copyright 2022, John Wiley and Sons. c) Schematic of the measuring configuration for MAPbI_3_ NWA PD to achieve visible light image sensing. Reproduced with permission.^[^ ^147^ ^]^ Copyright 2023, John Wiley and Sons. d) Schematic of a flexible image sensor. Reproduced with permission.^[^ ^35^ ^]^ Copyright 2016, John Wiley and Sons. e) e1: Layer‐by‐layer structure of 32 × 32 MAPbI_3_ NW image sensor. e2: Original and imaged love heart pattern. Reproduced with permission.^[^ ^186^ ^]^ Copyright 2016, John Wiley and Sons. f) f1: The working mechanism of electrochemical eye. f2: The reconstructed image of electrochemical eye and its projection on a flat plane. Reproduced with permission.^[^ ^22^ ^]^ Copyright 2020, Leilei Gu et al., published by Springer Nature. g) Detection mechanism of the area array PD and imaging result of MAPbI_3_ NWA PD. Reproduced under terms of the CC‐BY license.^[^ ^175^ ^]^ Copyright 2025, Gangjian Hu et al., published by Springer Nature. h) Schematic of the polarization imaging measurement system. Reproduced with permission.^[^ ^36^ ^]^ Copyright 2021, American Chemical Society. i) i1: Schematic of test device. i2: 2D gray scale under unpolarized light, p‐polarized light, and s‐polarized light. Reproduced with permission.^[^ ^151^ ^]^ Copyright 2021, John Wiley and Sons.

Lv et al.^[^ ^231^ ^]^ utilized a stepper motor to control the movement of a mask, sequentially constructing image pixels based on the response of an individual NW, thereby achieving imaging (**Figure** **17**a). Xu et al.^[^ ^229^ ^]^ also employed a single gradient bandgap MAPbX_3_ MW for spectral recognition and color imaging. The colorful imaging result was superimposed of a series of single‐wavelength induced current mapping and closely matched the target (Figure 17b). Xiong et al.^[^ ^147^ ^]^ designed a 7 × 7 pixel array using triangular NWA fabricated by the film confinement method as optical image sensors. They projected the feature image “T” onto the sensor using an optical mask, and the unblocked regions exhibited distinct responses. The detected feature image can be distinguished from the background signal by reflecting the switching state currents of all pixels (Figure 17c). Deng et al.^[^ ^170^ ^]^ developed an addressable PD array of 10 × 10 NWA by using the blade‐coating method, which also demonstrated good imaging capabilities. They further showcased the potential of a 21 × 21 flexible image sensor fabricated by blade‐coating on a PET substrate (Figure 17d).^[^ ^35^ ^]^ Gu et al.^[^ ^186^ ^]^ created a vertical NWA image sensor with 1024 pixels using an AAO template. They projected optical patterns onto the effective region of the sensor, and the image sensor function could be checked by addressing specific pixels by selecting the corresponding column and row numbers (Figure 17e). NWs grown by the AAO template are difficult to introduce cracks when bending,^[^ ^160^ ^]^ and even spherical devices can be fabricated.^[^ ^193^ ^]^ Gu et al.^[^ ^22^ ^]^ created a biomimetic eye with a hemispherical perovskite NWA retina, featuring a 10 × 10 FAPbI_3_ NWA PD array. This device can quickly reconstruct the patterns projected onto it. The low resolution of the patterns was mainly caused by the low density of liquid metal fibers (Figure 17f). Long et al.^[^ ^199^ ^]^ further endowed the biomimetic eye with filter‐free color imaging and neuromorphic preprocessing capabilities. Hu et al.^[^ ^175^ ^]^ demonstrated the advantages of MAPbI_3_ NW detectors in weak‐light imaging. The area array PD was exposed to monochromatic light at 365 nm with an optical power density of no more than 54 nW cm^−2^. Thanks to the excellent weak light detection capability of NWA, clear images were obtained under such weak light conditions (Figure 17g). Zhao et al.^[^ ^36^ ^]^ employed chiral 2D (R‐/S‐α‐PEA)_2_PbI_4_ NWA for polarization imaging. The measurement system included a light source, a linear polarizer, a quarter‐wave plate, a patterned photomask, and perovskite NW devices. The system exhibited good image fidelity and showed significant intensity differences under LCP and RCP illumination (Figure 17h). Song et al.^[^ ^151^ ^]^ developed a 24 × 24 integrated array of polarization PDs based on a Moiré lattice perovskite nanograting. The device exhibits different current distributions in patterns under illumination with unpolarized light, parallel polarized light (p‐polarized light), and perpendicular polarized light (s‐polarized light). This provides a new possibility to fabricate low‐cost and integrated polarization PDs for image application (Figure 17i).

## 6 Summary and Outlook

In conclusion, we review the existing reports on the synthesis of metal halide perovskites and their applications for PDs, focusing on four main areas: the synthesis of perovskite NWs and arrays, modification methods to improve NW performance, the performance of NW PDs, and applications based on PDs. In Chapter 2, we summarize the main synthesis methods for metal halide perovskite NWs and arrays, categorized into template‐free and template‐assisted approaches according to the synthesis ideas. Solution‐based methods, such as solution crystallization and colloidal methods, can be employed, as well as vapor deposition methods. Additionally, direct patterning of perovskites is another approach. Template‐assisted methods typically result in perovskite NWA with strict alignment, precise positioning, and homogeneous sizes, offering better uniformity and reproducibility compared to randomly distributed NWs, making them more suitable for large‐scale industrial production. There are also different strategies for controlling the morphology of perovskites using templates, including imprinting pre‐formed films to obtain NWs or inducing capillary force to drive the solution into templates, or growing in templates through vapor‐phase methods. Perovskite NWs that have not been specially treated often have room for performance improvement. Therefore, in Chapter 3 we summarize several modification methods aimed at improving NW performance or achieving specific properties, such as constructing perovskite HJT, adding special materials or encapsulating to improve performance and stability, and using special perovskite materials such as 2D layered perovskites or chiral perovskites. In Chapter 4, we introduce the concept of PDs and their key performance parameters. It is worth noting that the responsivity of NW PDs is usually significantly enhanced under weak light, which is one of the key features of NWs. As a result, there are some differences compared with traditional PDs. We summarize the performance parameters of several reported NW PDs and analyze some of the highlighted data. Finally, in Chapter 5, we discuss three important application directions based on NW PDs: polarization applications, flexible devices, and image sensing. These applications demonstrate the vast potential of perovskite NWs in photodetection.

However, despite significant advances in the synthesis and application of perovskite NWs, there are still areas with future promise. NW synthesis and device fabrication are more complex compared to thin films and single crystals. NWA synthesis often relies on high‐precision templates, which increases fabrication costs. Additionally, fabricating ultralong, continuous NWs without fractures requires high process precision. Maintaining high continuity and crystal quality over large areas in NWA is difficult, which limits large‐scale, high‐throughput industrial applications. Due to the extremely small dimensions of NW devices, some studies have focused solely on the peak performance of individual high‐performing devices with very small active areas, while overlooking average performance as well as potential errors introduced by hysteresis effects and measurement methods. This may lead to misleading conclusions in the research results. Furthermore, due to their small width, 1D NWs are challenging to fabricate into vertical devices, limiting many application directions. High‐quality vertical NWA can be fabricated using AAO templates, but it is difficult to obtain high responsivity and detectivity due to the limitation of the template. Perovskite NW structures are also more fragile, and they degrade quickly under high light intensity or harsh environmental conditions. Moreover, two major challenges that inevitably confront all perovskite materials are their long‐term stability and lead toxicity. The intrinsic instability of perovskites makes it difficult for optoelectronic devices to operate over extended periods, which has significantly hindered their commercialization. Although some devices with operational stability exceeding one year have been reported, this is still far from meeting the requirements for practical commercial PDs. Moreover, the presence of toxic lead in perovskites has further impeded their commercial adoption. In recent years, research on lead‐free perovskites has continued steadily; however, their performance remains far inferior to that of lead‐based perovskite materials to date. In conclusion, research on perovskite NWs should leverage the unique advantages of NW structures in weak light detection, polarization, and flexible devices, while continuously overcoming the challenges related to fabrication processes and stability. This requires collaborative efforts from both the academic and industrial sectors.

## Conflict of Interest

The authors declare no conflict of interest.
